# Immunotherapy: Examining Strategies to Address Cancer Complexity

**DOI:** 10.3390/ijms27219930

**Published:** 2026-11-06

**Authors:** Adrián Povo-Retana, Javier Ruiz-Navarro, Lucía Fernández-Frías, Carlota Alvarez-Lucena, Rodrigo Landauro-Vera, Manuel Izquierdo, Lisardo Boscá

**Affiliations:** 1 Instituto de Investigaciones Biomédicas Sols-Morreale (CSIC-UAM), Arturo Duperier 4, 28029 Madrid, Spain; javierruiz@iib.uam.es (J.R.-N.); luciaffrias@gmail.com (L.F.-F.); clucena@iib.uam.es (C.A.-L.); rlandauro@iib.uam.es (R.L.-V.); mizquierdo@iib.uam.es (M.I.); 2 Centro de Investigación Biomédica en Red de Enfermedades Cardiovasculares (CIBERCV), Institute of Health Carlos III (ISCIII), 28029 Madrid, Spain; 3 Faculty of Experimental Science, Universidad Francisco de Vitoria, Pozuelo de Alarcón, 28223 Madrid, Spain

**Keywords:** tumor microenvironment, CAR-T cells, engineered macrophages, immunotherapy, molecular oncology

## Abstract

Understanding the interplay between immune and stromal cells in the tumor microenvironment is essential for developing effective cancer therapies and preventing relapses. In this review, starting with the basics of tumor immunology and a historical review from cancer chemotherapy to current immunotherapy, in the context of precision medicine, we focus on immunotherapies based on genetically modified macrophages, and chimeric T-cell antigen receptor (CAR), highlighting the similarities and differences between TCR-endogenous signaling and CART-dependent signaling at the immune synapse, presenting all the generations that have been developed briefly, followed by NK cell-dependent CAR therapeutic approaches where a comparison is established, analyzing their respective advantages and drawbacks. Targeting the tumor-immune microenvironment has become central to cancer treatment, with combinatorial therapies applied and assessed as a feasible strategy for this complex group of diverse pathologies. In this context, modulating the immune synapse, rewiring immune cell metabolism, and developing genetically engineered immune cells have emerged as promising approaches that open novel avenues for treating and managing cancer patients. This review offers a holistic, historical view of state-of-the-art molecular approaches, along with insights into future perspectives and current limitations of the technologies supporting immunotherapy in molecular oncology.

## 1. Introduction

### The Immune System as a Central Hub in Coordinating an Anti-Tumor Response

Immunity encompasses all the organs, specialized tissue structures, cells, subcellular organelles and molecules that coordinate a response to protect the organism against pathogens, foreign substances, damaged cells, or any other insult that is recognized as a threat. This coordinated process is called the immune response, and it is formally divided into two stages based on the timing of molecular activation, duration, specificity, diversity, and ability to generate memory upon reencountering the pathogen. Thus, the immune response is classified as innate or adaptive [1].

The immediate and early immune responses (innate immunity) constitute the first line of defense. They include physicochemical barriers (skin and epithelial mucosa), antimicrobial by-products produced within these tissues, phagocytic cells (neutrophils, monocytes/macrophages, and dendritic cells), mast cells, basophils, eosinophils, natural killer cells, and innate lymphoid cells, along with blood proteins that complement this response and mediate inflammation.

The secondary response generated by lymphocytes depends on biomolecule production and is known as adaptive, or acquired, immunity. Essentially, it relies on two cell types: B-cells, which mediate humoral immunity, and T-cells, which mediate cellular adaptive immunity.

Natural killer (NK) cells are innate lymphocytes that exhibit cytotoxic activity against virus-infected cells and other cells infected by certain intracellular pathogens [2]. They kill infected cells through several mechanisms, including direct recognition and the absence of inhibitory signals present on non-infected cells, which culminate in the secretion of lytic proteins (granzyme and perforin) and mediate antibody-dependent cellular cytotoxicity via Fc-binding receptors. In addition, they produce interferon (IFN)-γ, which activates macrophages. Furthermore, these cells kill tumor cells without human leukocyte antigen (HLA) restriction or prior sensitization [3], playing a role in cancer immunosurveillance [4].

**Figure 1:**
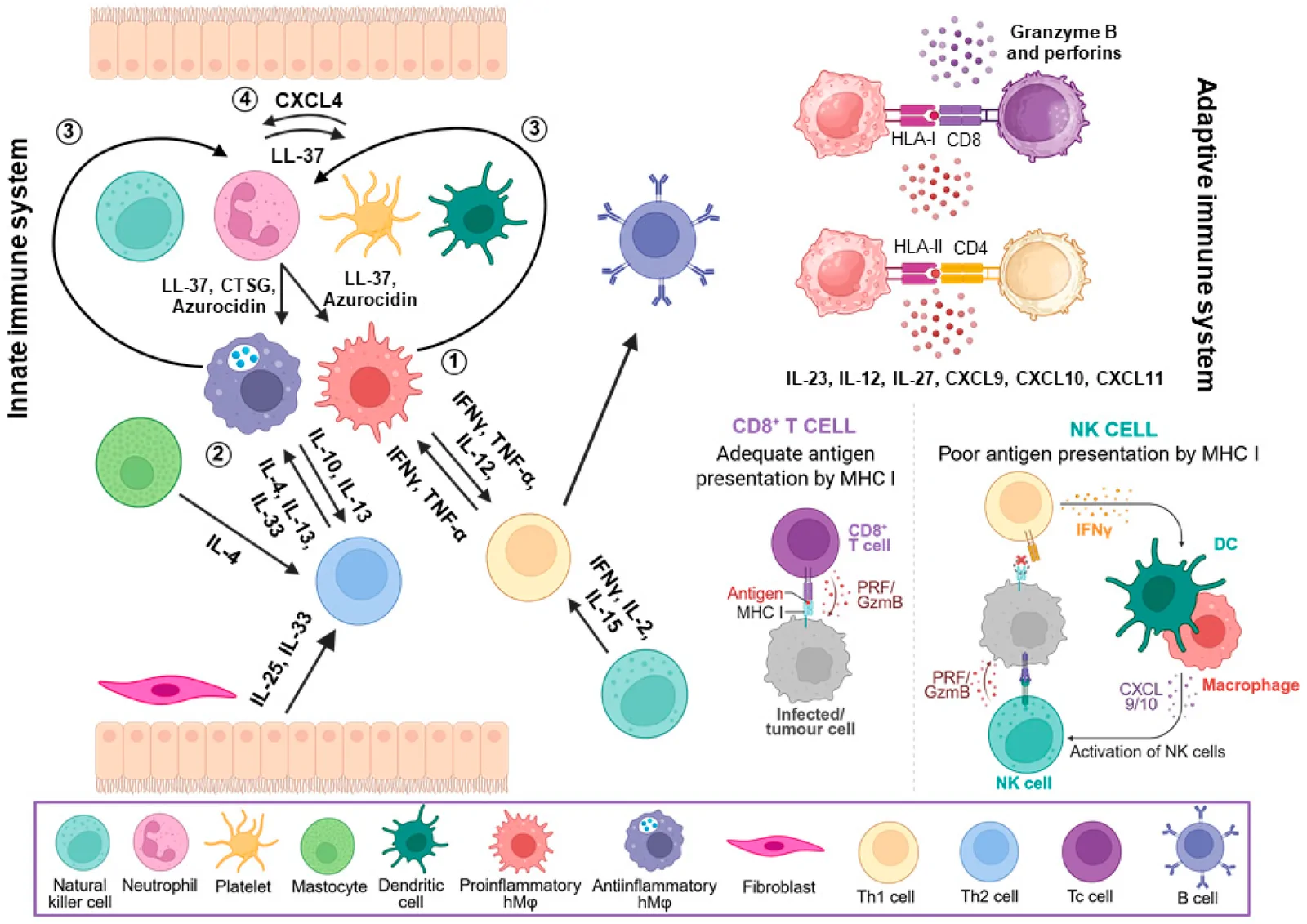
Coordinated response between the innate and adaptive immune systems. Tissue-resident macrophages become activated by PAMP or/and DAMP sensing signals by PRRs. ROS production and phagocytosis are activated, along with innate immune infiltration and accessory complement activation; an inflammatory response occurs. Th cells enhance the phagocytic phenotype (1). Once the threat or immune insult has been neutralized, a pro-resolution, tissue-regenerating anti-inflammatory response is activated after the acute phase. It relies on macrophage cytokine and growth factor production (IL-4, IL-10, IL-13, VEGF) and stromal non-immune stroma activation (IL-25 and IL-33 production) by fibroblasts and endothelial cells (2). Both pro-inflammatory and anti-inflammatory macrophages regulate neutrophil functional activation that infiltrates the tissue (3 and 4). APCs migrate to secondary lymphoid organs, where they present exogenous peptides on HLA-I to CD8^+^ T-cells or on HLA-II to CD4^+^ T-cells, inducing clone-specific proliferation and activation and generating effector T-cells that recirculate via lymphatics and the bloodstream. CD8^+^ T-cells will exert a direct cytotoxic effect on infected or tumoral cells that present infection-related peptides or neoantigen tumoral peptides (8–11 amino acids long) in HLA-I, whereas T helper cells will proliferate and be activated by APC-HLA-II peptide presentation and will either coordinate a Th1-dependent macrophage pro-inflammatory and phagocytic enhancement functional polarization or a Th-2 antibody production by B-cells, facilitated by infiltrated eosinophils, basophils and tissue-resident activated mast cells. NK cells exert direct cytotoxicity by activation in the absence of inhibitory signals, such as HLA-I tumor downregulation (immunoediting). Cross-presentation is much less frequent, and it occurs mainly by DC activation and, to a lesser extent, by macrophage activation. This figure was created in BioRender. https://BioRender.com/jcr0gpe (accessed on 23 July 2026).

Understanding the interplay between immune cells and how they coordinate responses is essential for developing targeted therapeutic approaches to tackle cancer and avoid undesirable side effects, as well as for restoring homeostatic immunosurveillance to prevent cancer relapse (Figure 1). Immunosurveillance is related to the ability that immune cells exert to recognize tumor cells at early stages of tumorigenesis and to eliminate them via cytotoxic effector mechanisms. This concept typically applies to lymphocytes, but it can extend to other cell types, such as macrophages.

## 2. The Adaptive Immune System and Interaction with the Innate Immune Response

Lymphocytes are generated in the bone marrow from hematopoietic stem cells. These cells undergo maturation processes within primary lymphoid organs: the bone marrow and thymus [5,6,7]. After maturation, they migrate to secondary lymphoid organs such as ganglia and spleen, where they proliferate and differentiate into effector cells and, eventually, into memory cells [8].

T-cells are the most abundant lymphocyte type and are characterized by TCR/CD3 expression. As mediators of cellular immunity, they recognize specific antigens presented via HLA-I/-II by antigen-presenting cells (APCs) to the T-cell receptor (TCR), and if recognition is productive, T-cells activate, provided secondary co-stimulatory signals and a cytokine-mediated response occur [9]. T-cell precursors migrate from the bone marrow to the thymus and into the thymic cortex, where they undergo positive selection, which preserves T-cells whose TCRs effectively recognize and bind to self-HLA molecules. Negative selection occurs in the corticomedullary junction and eliminates T-cells that strongly recognize self-antigens. The remaining cells differentiate into either CD3^+^CD4^+^ helper T (Th) lymphocytes or cytotoxic CD3^+^CD8^+^ lymphocytes [7]. In addition, most T-cell precursors that neither positive selection rescues nor negative selection eliminates die by neglect [10].

Th lymphocytes regulate cellular cytotoxic response and B-cell antibody production humoral response [11]. Th cells must bind to a specific antigen presented by a macrophage, dendritic cell, or B-cell (APCs) via HLA-II. These cells can differentiate into diverse subpopulations in response to stimuli, such as cytokines or transcription factor activation, which in turn promote the production of distinct soluble-molecule patterns. In addition, these cells exhibit functional phenotypic plasticity depending on the context [12,13], and can polarize into Th1, Th2 and other functional phenotypes (Th9, Th17, fTh).

Th1 differentiation is induced in response to intracellular microorganisms by IFN-γ and IL-12, which are produced by macrophages, dendritic cells and natural killer cells (DCs and NKs, respectively). The main function of Th1 is to coordinate the phagocytic-mediated defense by activating macrophages towards a proinflammatory phenotype [14].

Th2 lymphocytes differentiate in response to IL-4 secreted by mastocytes, and IL-25 and IL-33, which are produced by epithelial cells in response to cell damage caused by helminths or other extracellular parasites. They coordinate immune responses by activating eosinophils, mast cells that bind to IgE, as well as basophils, which have been identified as playing a critical role in allergy [14,15].

Cytotoxic T CD3^+^CD8^+^ lymphocytes recognize and eliminate infected and tumor cells [16] by specifically recognizing peptide-antigens presented in HLA-I receptors of target cells. This recognition promotes T-cell activation and enhances their cytotoxic functional capabilities, releasing granzymes and perforins, and inducing apoptosis via these soluble proteins and Fas ligand (FasL) [17,18,19,20,21,22]. In addition, NK cells can induce this cell death mechanism. CD3^+^CD8^+^ lymphocytes help maintain the Th1/Th2 balance through cytokine secretion.

B-cells are adaptive immune cells that mount the humoral response by antibody production and secretion [23], regulate DC function, and may act as APCs when they present antigens to T-cells.

Macrophages are innate immune cells that perform multiple and essential biological functions. These cells are phagocyte specialists and bridge innate and adaptive immunity by acting as antigen-presenting cells that prime T-cells. Macrophages potentiate innate immunity and instruct the adaptive response [24]. The innate response is amplified by the secretion of proinflammatory mediators and chemokines that target several cell types, including monocytes, other macrophages, T-cells, B-cells, NK cells, neutrophils, and epithelial and endothelial cells. This myeloid subset has become increasingly crucial in cancer patient outcomes; modulating the immune response at the functional level to facilitate tumor remission/clearance is a major area of biomedical research.

Neutrophils and monocytes are among the first cell types to be recruited in an inflammatory response from the bloodstream and once they become activated by pattern recognition receptors’ interactions with pathogen-associated molecular patterns or damage-associated molecular patterns. They release several mediators (macrophages: IL-1β, IL-6, TNF-α; neutrophils defensins such as LL-37/LL-39 [25,26], cathepsin G, azurozidin, etc.). Sentinel tissue-resident cells, as well as non-immune stromal cells such as endothelial cells, are activated and produce IL-33 and IL-25 cytokines [27,28,29]. APCs then migrate to secondary lymphoid organs to present antigens and activate T-cells, which in turn orchestrate the adaptive response. Upon viral infection, NK cells, DCs and macrophages produce IFN-γ, favoring the overexpression of HLA-I/HLA-II molecules, as shown in Figure 1. Alternative activation occurs once the pathogen has been neutralized; there is a transition from the acute inflammatory phase to the resolution phase, reaching a homeostatic physiological state.

## 3. The Chemotherapy Timeline in Cancer and Immunotherapy

**Figure 2:**
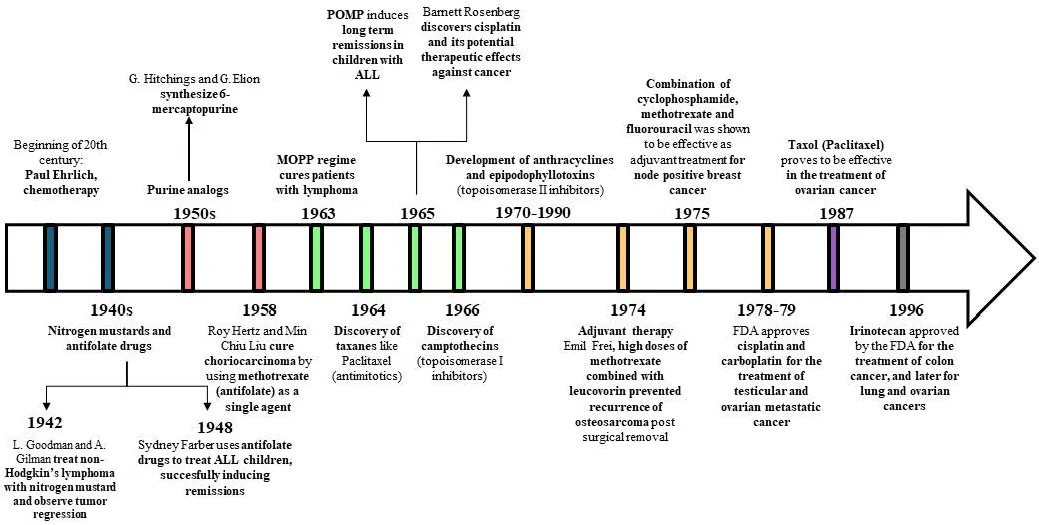
Oncological chemotherapy timeline. This figure summarizes pivotal discoveries and drug development in molecular oncology.

The concept and use of chemotherapy were established at the beginning of the 20th century by Paul Ehrlich, who defined it as “the use of chemical products to treat diseases” [30,31], initiating an area in the treatment of cancer that extends to this day, with continued advances in the development of drugs against cancer [32]. Figure 2 shows a schematic timeline of progress in cancer chemotherapeutic drug development.

Success was not observed until the end of the 4th decade of the 20th century. In fact, the main limitations in anticancer drug discovery were the absence of adequate models that could be effectively used to narrow the vast number of simple molecules to a reduced group displaying anticancer activity in humans, and the limited development of cancer therapy outside the clinical setting to allow testing of potential therapeutic molecules (https://www.cancer.gov/research/progress/250-years-milestones (accessed on 23 July 2026)).

In cancer therapy, the 1940s saw the discovery and initial use of antifolate drugs and nitrogen mustards. In 1942, L. Goodman and A. Gilman observed tumor regression after treating a patient with non-Hodgkin lymphoma using nitrogen mustard. Although this remission only lasted for a few weeks, the experiment demonstrated that systemic administration of such drugs could induce tumor regression. Subsequently, in 1948, as a second approach to cancer drug therapy, S. Farber introduced antifolate drugs—first aminopterin, then amethopterin (methotrexate)—to treat children suffering acute lymphoblastic leukemia (ALL), successfully inducing remission. Methotrexate as a single agent exhibited anti-tumor activity. However, the most notable effects of methotrexate were not observed until 1958. It was successfully applied as a single agent to treat choriocarcinoma. Furthermore, in 1974, Emil Frei combined high doses of methotrexate with leucovorin to prevent recurrence of osteosarcoma after surgical removal, obtaining promising results and laying the groundwork for adjuvant therapy. Later, in 1975, the combination of cyclophosphamide, methotrexate, and fluorouracil, known as the CMF regimen, was developed and proven effective as one of the first adjuvant chemotherapy treatments for lymph node-positive breast cancer. In the mid-1960s, a broad program established by Zubrod for the collection and testing of natural products, such as plant and marine sources, led to the discovery of taxanes and camptothecins [33,34].

The application of platinum derivatives for cancer treatment marked a significant milestone; however, a major limitation of these compounds is their associated organ toxicity. Taxol derivatives and topoisomerase inhibitors are now being combined with other anti-tumor agents and immunotherapy to harness their tumor-inhibiting properties while reducing standard doses to minimize side effects [35]. The concept and use of chemotherapy were established at the beginning of the 20th century by Paul Ehrlich, who defined it as “the use of chemical products to treat diseases” [30,31], initiating an area in the treatment of cancer that extends to this day, with continued advances in the development of drugs against cancer [32]. Figure 2 shows a schematic timeline of progress in cancer chemotherapeutic drug development.

The US national drug development effort was carried out through the Cancer Chemotherapy National Service Center, established in the mid-1950s. In oncology, the concept of a cancer cure was introduced in the 1960s with the recognition of medical oncology as a clinical specialty [30]. In addition, in most medical centers, chemotherapy was considered the last option for cancer patients because most anticancer drugs caused more harm than good.

The success of drug treatment was considered incompatible with preserving physical health because the drugs were extremely aggressive. Perhaps the use of the term “poison” to refer to chemotherapy contributed to devaluing the development of cancer drug research. As a major milestone in cancer chemotherapy, the success in curing acute childhood leukemia and advanced Hodgkin’s disease using a combination of drugs during the transition from 1960 to 1970 was instrumental in changing opinions about the effectiveness of drugs in curing advanced cancers [36]. This success enabled research into adjuvant chemotherapy and helped launch the U.S. National Cancer Program.

Chemotherapy research rapidly expanded, and within a few years, the complex MOMP program (from the acronym for the combination of mechlorethamine, vincristine, melphalan, and prednisone) for the treatment of Hodgkin’s disease changed to MOPP after replacing melphalan with procarbazine. Despite resistance from regulatory authorities, success in treating these patients was remarkable, with the complete remission rate increasing from almost zero to 80%. In addition, Hodgkin’s patients who achieved a MOPP-dependent complete remission never relapsed with a follow-up of more than 20 years [37].

The advent of monoclonal antibodies (antibodies with one specificity in the Fab fraction), including checkpoint inhibitors, and cell-based therapies leveraging chimeric antigen receptors (CARs: CAR-macrophages, CAR-T, CAR-NK, and other innovative strategies) has improved the effectiveness of simple molecules and promoted research in the field of chemotherapy [38,39]. Hybridomas, described in 1975, and monoclonal antibodies were successfully introduced in the mid-1990s to treat different types of cancer; they are generated by immunization and B-cell splenic isolation and ex vivo melanoma fusion, and each clone exhibits a single-epitope specificity. This finding revolutionized the molecular immunology field, giving rise to a potent industry that has benefited millions of patients worldwide [40]. Interestingly, combining antibodies with chemotherapy proved highly effective. This is the case for cetuximab and bevacizumab in colorectal cancer, trastuzumab in breast cancer, and rituximab in non-Hodgkin’s lymphoma; each monoclonal antibody treatment protocol includes chemotherapeutic agents to treat these common tumors. For these reasons, the development of combinations of new biological and active cytotoxic agents keeps increasing and the number of cancers successfully treated with combined modalities of therapies is expected to rise [41,42,43].

As a result, the cancer mortality trend has been declining continuously since the 1990s, partly due to prevention policies such as smoking restriction, new diagnostic tools, and prognostic biomarkers, but also due to advances in cancer therapeutics, largely associated with the inclusion of chemotherapy in most treatment protocols [44,45,46]. Today, in the era of precision medicine, chemotherapy has changed as researchers use the identification of specific molecular alterations that lead to cancer to screen for potential new drugs and treatments. These molecular specificities range from mutations in BRAF and RAS to other mechanisms that promote metabolic alterations, such as mutated isocitrate dehydrogenase in gliomas and glioblastomas, which results in an imbalance in α-ketoglutarate and renders 2-hydroxyglutarate, a competitive inhibitor of α-ketoglutarate-dependent dioxygenases, which is considered an oncometabolite [47,48]. 2-hydroxyglutarate alters the transcriptome, leading to changes in histone acetylation and methylation. Hence, specific mutations in isocitrate dehydrogenase can be targeted with impressive therapeutic results [49,50].

Modern immunotherapy relies on molecular knowledge; however, the first reported case of immunologic human therapeutic intervention occurred in China around 1000 BC. Chinese practitioners dried the crusts from pustules, pulverized them, and made children inhale the dust. This airway-dependent administration was called variolation. These practices reached Constantinople (modern-day Turkey), where the dust was injected into the skin. This method did not go unnoticed by Marie Wortley, who attempted to introduce it in Great Britain without success, despite its beneficial immunization effects. Years later, Jenner conducted the first systematic attempt to vaccinate the population by inoculating James Phipps with pulverized cowpox (vaccine virus) lesion material and demonstrated its protective effect. His work, published in 1798, initially faced rejection, but it laid the foundation for vaccination [51,52].

Jenner [53,54,55] and Virchow and Ehrlich paved the way with smallpox vaccination and the observation that inflammation and cancer were related and the hypothesis that tumors might be controlled by the immune system [56,57].

**Figure 3:**
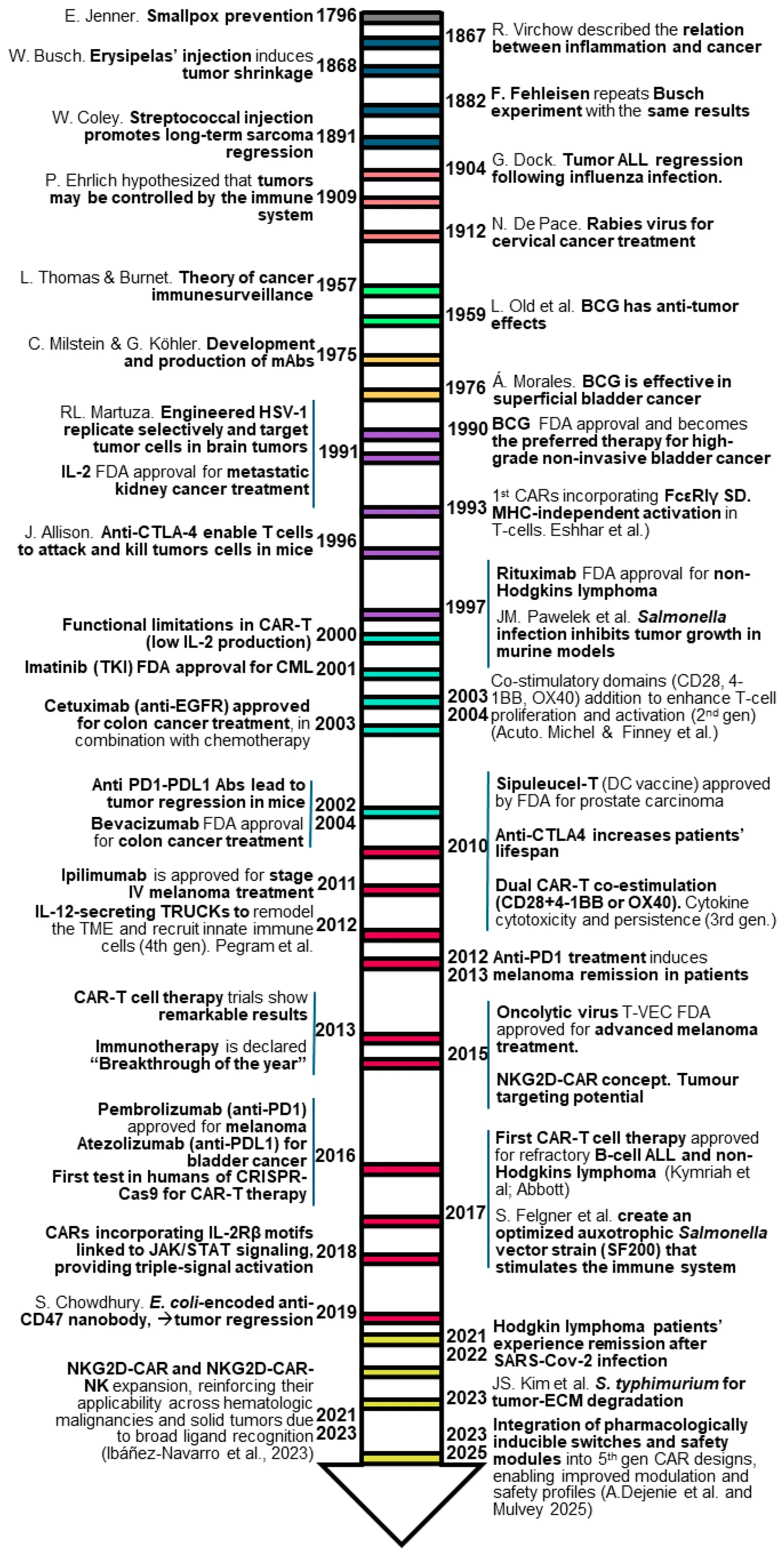
Timeline of molecular immunotherapy from a historical and functional perspective. This figure summarizes pivotal milestone discoveries in molecular oncology, molecular immunology, and related therapies from 1796 to the present day.

In the late 19th and early 20th centuries, advances in microbiology and the discovery of innate immunity by Illya Mechnikov dramatically increased therapeutic possibilities. The idea of activating the immune system by the challenge with infectious pathogens was not new. W. Busch [58] reported tumor shrinkage by erysipelas injection, Coley et al. observed long-term sarcoma regression [58], Dock et al. documented ALL regression after influenza infection [59], and in 1957 Thomas and Burnet postulated the theory of immune surveillance in cancer [57]. Shortly after BCG exerted anti-tumor capacity and proved to be effective against bladder cancer, and was finally approved by the FDA in 1990 [57,58,59], as well as engineered viruses [57] (Figure 3). Within this period, molecular medicine and precision therapy experienced one of the most groundbreaking discoveries: the development and monoclonal antibodies in 1975, which granted Milstein and Köhler the Nobel Prize in Medicine and Physiology nine years later, in 1984 [60,61,62]. This discovery marked the beginning of the modern immunotherapy era. It opened novel, unexplored and unprecedented venues for cancer treatment: anti-CTLA-4 and anti-PD-L1 enabled T-cells to attack tumor cells in mice [20,34,57,58]. Retuximab was approved by the FDA in 1997 and Cetuximab and bevacizumab followed the same path for colon cancer in 2003 and 2004, respectively.

There are a few more examples within the timeline that evidence the enormous impact of monoclonal antibodies in the field (Ipilimumab and pembrolizumab for melanoma, and atezolizumab for bladder cancer) [58]. Simultaneously, a plethora of specific inhibitors (-inibs’) and dendritic cell-based vaccines have been developed. Immunotherapy was declared the breakthrough of the year in 2013.

Starting in 2017, genetic engineering technologies took over; this group includes oncolytic viruses, antibody-producing bacteria, and CAR technologies involving T and NK lymphocytes as well as macrophages. At the dawn of this millennium, CAR-T lymphocytes were first generated (2003–2017), demonstrating spectacular results in the treatment of hematological tumors.

In 1993, the first CARs incorporating CD3ζ or FcεRIγ signaling domains were developed, enabling HLA-independent activation of T lymphocytes (first generation) [63].

In 2000, the functional limitations of first-generation CARs were confirmed, including insufficient activation, low IL-2 production, and reliance on exogenous IL-2 supplementation [64]. In 2003–2004, co-stimulatory domains (CD28, 4-1BB, OX40) were introduced to enhance T-cell proliferation, persistence, and effector function (second generation) [65,66]. CAR constructs incorporating dual co-stimulation, such as CD28 plus 4-1BB or OX40, were designed to augment cytokine production, cytotoxicity, and persistence (third generation; 2010). In 2012, inducible IL-12-secreting T-cell Redirected for Universal Cytokine Killing (TRUCKs) were developed, enabling CAR-dependent cytokine release to remodel the tumor microenvironment and recruit innate immune components (fourth generation) [67]. In 2015, the NKG2D-CAR concept was consolidated, recognizing the receptor’s ability to detect multiple stress-induced ligands and broaden tumor-targeting potential (NKG2D-CAR lineage).

Between 2012 and 2016, comparative clinical assessments of second- versus third-generation CARs revealed increased signaling strength in third-generation constructs, as well as tonic signaling, accelerated exhaustion, and higher toxicity risks.

In 2017, the first FDA approvals were granted: CD19-targeting second-generation CAR-T therapies achieved durable remissions in B-cell ALL and lymphomas, establishing a clinical standard [68,69].

The introduction of CARs incorporating IL-2Rβ motifs linked to JAK/STAT signaling, providing triple-signal activation (CD3ζ + co-stimulation + JAK/STAT), emerged in 2018, thereby improving functional control and persistence (fifth generation [70]).

The expansion of NKG2D-CAR and NKG2D-CAR-NK platforms, reinforcing their applicability across hematologic malignancies and solid tumors due to broad ligand recognition, took place between 2020 and 2023 [71].

Finally, the integration of pharmacologically inducible switches and advanced safety modules into fifth-generation CAR designs enables refined modulation of in vivo CAR-T activity and improved safety profiles, which have recently been developed (2022–2025) [72,73] (Figure 3). It is becoming clear that genetic engineering technologies are powerful tools for creating the next generation of tailored precision medicine therapies, where interdisciplinary collaboration among clinicians and basic researchers from all fields is essential to address the complexity of various tumor pathologies.

## 4. Precision Medicine in the Field of Cancer

Precision medicine is already delivering treatments targeted at specific clinical conditions, including cancer diagnostics, prognostics, and therapy [74,75,76]. Advances in precision medicine, combined with risk prediction tools and increased therapy customization, are the foundation for evidence-based medical decision-making to improve clinical outcomes and prevent iatrogenic disorders, such as adverse effects of oncological chemotherapy on the hepatic, renal, cardiovascular, or neural systems. In this sense, the path of personalized therapy will advance significantly [77,78].

Furthermore, advances in cancer detection and accurate diagnosis, personalized therapy design, and powerful artificial intelligence are key to expanding precision medicine [79]. The concept of precision medicine, as defined by the NCI, is evolving to include precision diagnostics and individualized therapies to improve patient outcomes and reduce costs for health systems.

Indeed, precision medicine means designing personalized patient care based on individual genetic characteristics, and expecting responses to oncological treatments to be similar across these patients. Even though genetic data provide a strong rationale, they may ultimately be somewhat limiting, and additional information is required to be incorporated into the personalized medicine portfolio [75]. This idea has taken two forms: first, precision diagnosis, aimed at improving diagnostic accuracy, and second, reducing biased variation in pathological characterization. The concept aims at the generation of standardized “open-access” protocols to ensure more consistent diagnoses and treatments.

The second major pillar of precision medicine is the formulation of the concept of individualized therapy. This includes personalizing care where it matters, which involves treating patients based on the genetic profiles they fall into and treating them similarly. This allows earlier intervention in cases where patients do not respond to treatment. Additionally, precision medicine uses advanced therapies, including combined therapies that integrate immunotherapy, CAR cell-based therapies (CAR-T, CAR-NK, CAR-macrophages, etc.), and radiotherapy, to optimize efficacy, minimize errors, and reduce costs. Hence, individualized therapies leverage specific therapeutic approaches validated by independent groups.

In this area, artificial intelligence (AI) and machine learning (ML) enable validation of different protocols and identification of markers associated with specific tumor types, which could predict treatment response and prognosis. These tools fine-tune the assessment of the therapeutic impact of the treatments. Finally, the development of predictive analysis-based tools to assess disease progression risks offers opportunities for earlier intervention to benefit the patient. In this scenario, evidence-based criteria may identify new uses for existing compounds.

## 5. Artificial Intelligence in Precision Immuno-Oncology: Data Quality, Multimodal Integration and Responsible Clinical Translation

Artificial intelligence (AI) and machine-learning approaches are increasingly used in precision oncology to integrate high-dimensional multimodal molecular and clinical data, identify biomarkers, stratify patients, and support therapeutic decision-making. However, an AI model’s performance is inseparable from the quality, provenance, representativeness, and temporal relevance of the data it learns from. In rapidly evolving fields such as cancer immunotherapy, databases assembled under prior diagnostic criteria, sequencing technologies, treatment standards, or clinical workflows can encode patterns that no longer adequately represent the population or therapeutic context in which an algorithm is deployed. Therefore, the central challenge is not simply to increase data volume but to establish data ecosystems that are sufficiently current, standardized, traceable, representative, and clinically relevant enough to support reproducible inference. Because biomedical databases are heterogeneous and differ substantially in content, purpose, and validation procedures, no single universal proportion of “experimentally validated” observations can fully define data reliability. Instead, data fitness for purpose should be assessed according to the validity of source measurements and labels, completeness, provenance, metadata quality, representativeness and temporal relevance; model reliability must subsequently be established through independent external and, when appropriate, prospective validation [80,81,82]. These considerations are particularly important when AI-derived predictions might influence patient selection, biomarker interpretation, or immunotherapeutic strategies where both clinical and molecular information must be integrated in order to properly address tumor complexity.

## 6. Database Updating and Standardization

Developing clinically useful AI requires curated databases that evolve alongside biomedical knowledge and clinical practice. Heterogeneity can arise at multiple levels, including differences in patient populations, diagnostic criteria, sample processing, sequencing platforms, imaging acquisition, annotation procedures, treatment regimens, outcome definitions, and duration of follow-up. If these sources of variation are not explicitly represented, models may learn site-, platform-, or period-specific correlations rather than biologically or clinically transferable relationships. Public cancer datasets can additionally underrepresent specific demographic groups, and a recent computational pathology study demonstrated that such imbalances can translate into substantial differences in model performance between patient subgroups [83].

Consequently, database stewardship should be regarded as a continuous component of AI development rather than a one-time preprocessing step. Core requirements include harmonized definitions of clinical variables and endpoints; explicit documentation of assay and acquisition platforms; controlled terminologies and ontologies; longitudinal treatment and outcome annotation; quality-control metrics; versioning; and sufficiently rich metadata to reconstruct where, when and how each observation was generated [80,81].

The FAIR principles provide a widely adopted conceptual basis by requiring research data and metadata to be Findable, Accessible, Interoperable and Reusable, with explicit provenance and adherence to domain-relevant standards [80]. In oncology, initiatives such as AACR Project GENIE illustrate the value of harmonizing clinical-grade genomic and clinical outcome data across institutions, whereas the Minimal Common Oncology Data Elements (mCODE) initiative provides a consensus framework for representing interoperable oncology data [84,85].

Open-science and data-sharing initiatives should therefore be promoted together with standardized acquisition and reporting protocols. Importantly, “open” clinical data should not be equated with unrestricted release of individual-level health information. A more suitable framework combines open standards, transparent data dictionaries, reusable analytical code and, whenever it is both ethically and legally possible, de-identified datasets with governed-access or privacy-preserving mechanisms for sensitive patient-related information [80,86]. Such frameworks facilitate reproducibility and independent validation across institutions, population cohorts and therapeutic settings.

In precision immuno-oncology, where biomarker definition and standards of care evolve rapidly, regular database updating should additionally document changes in treatment exposure, molecular testing and outcome ascertainment to minimize the incorporation of obsolete clinical patterns into future decision-support systems [81,87].

## 7. Multimodal Data Integration

Cancer is intrinsically multidimensional, and no single data modality captures the full biological state of the tumor, its microenvironment and the host. Genomic data describe mutations, both somatic alterations and inherited variation; transcriptomic profiles capture context-dependent cellular programs; proteomic data provide information on protein abundance and signaling states; histopathological and spatial measurements resolve tissue architecture and the distribution of tumor, immune and stromal populations; radiological imaging captures macroscopic heterogeneity and disease burden throughout the body; and longitudinal clinical data provide treatment exposure, comorbidities, toxicity and patient outcomes. Hence, integrating these layers is particularly relevant to immunotherapy, where treatment response emerges from interactions among tumor-intrinsic features, immune-cell states, stromal in situ organization, systemic host factors, and prior therapy rather than a single molecular determinant.

Evidence from immuno-oncology provides a proof of principle for this approach. In a retrospective single-center cohort of 247 patients with advanced non-small-cell lung cancer treated with PD-L1 blockade, Vanguri et al. integrated radiological, pathological and genomic information and showed that multimodal models outperformed individual modalities: radiology plus genomics reached an AUC of 0.76, whereas three-modality integration incorporating the pathologist-assessed PD-L1 tumor proportion score reached an AUC of 0.80 [88]. Multimodal approaches have also combined whole-slide histology with molecular profile data across multiple cancer types, demonstrating that morphologic, spatial and molecular information can provide complementary prognostic information [89].

Nevertheless, such integration should not be assumed to automatically correct poor-quality data. Combining modalities does not eliminate modality-specific biases, batch effects, asynchronous sampling, or data lacking clinical or molecular meaning, and may even introduce them if they are not explicitly addressed. For example, tumor tissue obtained at diagnosis may not represent the tumor microenvironment (TME) after several lines of treatment, whereas radiological and circulating biomarkers can be collected repeatedly, reflecting different timeframes that drive disease and may influence treatment choice and modify prognostic outcomes.

Multimodal AI therefore requires accurate patient-level linkage, temporal alignment of measurements, harmonized preprocessing and explicit methods for handling missing modalities and uncertainty. When these requirements are met, multimodal analysis offers a biologically more coherent framework for personalized immunotherapy because it enables models to characterize the patient–tumor ecosystem rather than rely on a single, potentially incomplete biomarker panel.

## 8. Critical and Continuous Evaluation of AI Models

A model that performs well on its development dataset may not perform equally well in another hospital, patient population, or time period. Differences in prevalence, referral patterns, laboratory platforms, scanners, clinical workflows, and therapeutic standards can produce dataset shift, in which the distribution of model inputs, outcomes, or their relationship differs between development and deployment [87]. Dataset shift is particularly relevant in oncology because molecular classifications, treatment sequences, regulatory approvals, and biomarker testing practices are continuously evolving.

Clinical AI should therefore undergo validation as a lifecycle process, automated actualization and correction. Before implementation, independent external validation should assess performance across different institutions, populations, acquisition technologies and clinically relevant subgroups. After implementation, prospective surveillance should assess discrimination but also calibration, data quality, subgroup performance, uncertainty, safety, and evidence of temporal or distributional drift. Model and dataset versions should be traceable, and predefined procedures should determine when recalibration, retraining, revalidation, or suspension is required. International initiatives increasingly formalize these principles. FUTURE-AI addresses the complete healthcare-AI lifecycle through the principles of fairness, universality, traceability, usability, robustness, and explanation degree; DECIDE-AI emphasizes early clinical evaluation and the human factors surrounding AI use; and PROBAST+AI provides a structured framework for assessing methodological quality, risk of bias, and applicability of prediction models [81,82,90].

Explainable AI can contribute to this process by allowing developers and clinical users to interrogate which variables or image regions influence model output, identify biologically implausible associations and investigate potential failure modes. However, explanation degree must not be interpreted as evidence that a prediction is correct, causal or clinically safe; it should be used as a consideration for clinicians and researchers to correct, implement and train models based on the provided data or clinical facts that would have to be further interpreted by experts. Current explanation methods can themselves fail in ways that make patient-level explanations unreliable or misleading. Ghassemi et al. have therefore argued that explanation degree should not substitute for rigorous internal and external validation [91]. In clinical immuno-oncology, explanation degree is best considered an adjunct for model interrogation and audit, embedded within a broader framework of calibration, independent validation, and prospective evaluation.

## 9. Encourage Multidisciplinary Training and Team Collaboration

Data scientists cannot develop reliable clinical AI in isolation from the biological and clinical processes that generate the data. Clinicians are required to define the intended clinical use, relevant outcomes, acceptable risk, and the consequences of false-positive or false-negative predictions; molecular and cellular biomedical researchers are needed to assess whether candidate features and model associations are biologically plausible and to identify limitations of experimental assays; statisticians and data scientists contribute expertise in study design, missing-data mechanisms, model development, calibration and uncertainty; and AI and software engineers are essential for reproducible pipelines, interoperability, deployment, and post-implementation monitoring. This interdisciplinary structure is particularly important in immunotherapy, where molecular information must be interpreted in the context of tumor evolution, TME composition, therapeutic exposure and immune-mediated toxicity.

Training must also be bidirectional. Clinicians and experimental scientists need sufficient AI literacy to understand concepts such as model calibration, overfitting, distribution shift, uncertainty, and subgroup bias, whereas computational specialists need sufficient understanding of biological sampling and clinical workflows to recognize when technically valid inputs are clinically inappropriate. The FUTURE-AI consensus, developed by 117 interdisciplinary experts from 50 countries, explicitly recommends continuous engagement of interdisciplinary stakeholders throughout the AI lifecycle. DECIDE-AI likewise emphasizes the evaluation of human–AI interaction, user-related factors and integration into real clinical workflows during early clinical assessment [90].

For precision immuno-oncology, this collaborative model should extend from data acquisition to interpretation and clinical deployment. Multidisciplinary oversight can improve annotation quality, identify clinically inappropriate surrogate endpoints, detect biologically implausible correlations and determine whether an apparently accurate model provides information that is genuinely actionable beyond established biomarkers. Thus, multidisciplinary training is not simply an educational requirement; it is essential to data quality assurance and model validation.

## 10. Planning High-Quality Prospective Studies

Many healthcare AI systems are initially developed and evaluated retrospectively, an appropriate setting for methodological development and hypothesis generation but insufficient to establish clinical utility. Clinical deployment changes the context in which an algorithm operates: clinicians can respond to its recommendations, patient selection can change, and workflow or behavioral effects can influence outcomes. DECIDE-AI consequently emphasizes early evaluation under real clinical conditions, whereas SPIRIT-AI and CONSORT-AI extend established reporting standards to protocols and reports of clinical trials involving AI interventions, respectively [90,92,93].

The gap between technical performance and prospective clinical evidence remains relevant. A 2024-scoping review identified 86 randomized controlled trials evaluating AI integrated into clinical practice; 70 (81%) reported a favorable primary endpoint, but 54 (63%) were conducted at a single center and race or ethnicity was reported in only 22 trials, raising concerns about representativeness and generalizability [94].

Prospective AI studies in immuno-oncology should therefore use standardized and pre-specified data-collection protocols, clearly define the intended-use population and clinical decision, incorporate independent and preferably multicenter evaluation, and select endpoints that reflect clinically meaningful benefits rather than algorithmic accuracy alone. Longitudinal follow-up is particularly important because short-term tumor response does not capture durability, progression-free or overall survival, late toxicity, immune-related adverse events, or treatment sequencing. Prospective studies can simultaneously provide higher-quality datasets for future model development, provided specimens, imaging, molecular profiles, treatment exposures and clinical outcomes are collected using harmonized procedures. In this way, prospective evaluation can test clinical utility while generating higher-quality data for subsequent model development.

## 11. Development of Interoperable Data Infrastructures

Even high-quality datasets have limited value for generalizable AI if they remain isolated within individual institutions. Interoperability must therefore be addressed at several complementary levels: semantic interoperability through shared terminologies and ontologies; structural interoperability through common data models and exchange standards; methodological interoperability through harmonized definitions and analytical procedures; and governance interoperability through consistent rules for consent, access, provenance, privacy, and secondary use. Initiatives such as mCODE and community standards including FHIR, OMOP, DICOM and SNOMED CT provide mechanisms for reducing institution-specific representations of equivalent clinical information, and FUTURE-AI explicitly recommends the adoption of established community-defined standards to improve interoperability and transferability across clinical settings [81,85].

For molecular data, the Global Alliance for Genomics and Health (GA4GH) has developed interoperable technical standards and policy frameworks intended to support responsible international sharing of genomic and related clinical information through both harmonized aggregation and federated approaches [86]. These approaches matter because meaningful cross-center learning does not require unrestricted centralization of raw patient data. Distributed-learning approaches can reduce the need to pool raw patient-level information centrally. As a proof of principle, Swarm Learning has demonstrated decentralized machine learning across distributed clinical datasets, including blood transcriptomic data from patients with leukemia, without centralized pooling of the underlying datasets [86].

Interoperable infrastructures not only should ultimately enable model training but also independent multicenter validation, continuous performance monitoring and comparison of therapeutic strategies across heterogeneous real-world populations. Privacy and security must remain integral to this architecture through appropriate governance, access controls, encryption and other privacy-enhancing technologies [81,86]. Thus, the objective is not to maximize data centralization, but to create a distributed learning ecosystem that can analyze and validate clinically relevant information across institutions while protecting patient privacy and meeting applicable data-governance requirements.

These challenges have direct ethical consequences. In precision immuno-oncology, biased, obsolete, or poorly validated models could influence biomarker interpretation, patient eligibility, treatment selection, toxicity assessment, or prognosis; this is why human intervention in this process remains crucial, essential, and irreplaceable. A systematic performance disparity may therefore translate into disparities in clinical opportunity or harm when algorithmic output enters the therapeutic pathway. The documented demographic performance differences in computational pathology illustrate that fairness cannot be considered solely a technical property of the algorithm; it also depends on how data are collected, whose data are represented, how models are evaluated across subgroups, and where they are deployed [81,83].

Accordingly, data governance, prospective validation, continuous monitoring and clinician education should be considered patient-safety requirements rather than ancillary technical tasks. Clinicians using AI-supported systems should understand their intended use, validation population, uncertainty and recognized failure modes, while developers and institutions should maintain transparent mechanisms for performance auditing and corrective action [81,87]. AI has considerable potential to support increasingly personalized immunotherapeutic strategies, but its clinical value will ultimately depend on the quality and representativeness of the evidence on which its predictions are based. Responsible implementation therefore requires that technological innovation proceed together with rigorous data stewardship, clinical validation and ethical accountability. Recognizing these limitations is important for understanding the constraints of the proposed trained models, as well as the possible final interpretations and conclusions that could directly impact patient management and outcomes.

## 12. Immunotherapy and Tumor Vaccines

Immunotherapy is defined as the use of molecules with biological activity, which are normally inhibitors, biomolecules (growth factors, checkpoint inhibitors, cytokines, monoclonal antibodies), vaccines (carrying tumor neoantigens), or any engineered or primed cell (CAR-T cells, CAR-NK cells, primed DCs, macrophages) that modulate the immune response to prevent or combat disease. Disease can have diverse origins: exogenous, caused by a pathogen (infection), or endogenous, due to immune dysfunction. This block mainly covers three categories: autoimmune diseases, chronic inflammation-related diseases, and cancer.

Vaccination has been a major breakthrough in modern medicine. Immunization relies on the immune system’s ability to store information (memory) once it has encountered the antigen that elicits an immune response [95]. The immunization provided by vaccines is estimated to save 2–3 million lives worldwide every year [95,96]. This type of active immunization has inspired the design of tumor vaccines. The earliest attempt to treat cancer by immunization was reported in 1891 by W.B. Coley, who injected streptococcal organisms into a patient and observed bone and tissue sarcoma regression [97,98]. Currently, prophylactic vaccines are available for cancer prevention. This is because their primary source is an infectious microorganism; hepatitis B virus can lead to hepatocarcinoma, Epstein-Barr virus can induce the beginning of nasopharyngeal carcinoma, human papillomavirus can give rise to cervical cancer, and *Helicobacter pylori* can cause gastric cancer [99]. Tumor vaccines are based on the fact that tumors express modified proteins arising from genomic instability and mutations; these novel antigens are commonly referred to as neoantigens. Meanwhile, physiological antigens that exhibit anomalous spatiotemporal expression within a tumor are called tumor-associated antigens; they are typically included in general biomarker panels, are increasingly used in daily clinical practice, and, under normal conditions, are expressed during development.

Neoantigens are a source of novel non-self targets for immune recognition. Several tools have been developed to identify these markers, including prediction approaches (bioinformatics), analytical observation (immunopeptidomics), and screening, which typically involves blood testing. The main approaches aim to activate DCs, and their formats and forms vary: short peptides can favor HLA-I recognition; mRNA or DNA vaccines can encode neoantigens; DAMPs, co-stimulatory molecules, and microbial or cellular vectors can ultimately direct the adaptive immune system and promote T-cell and B-cell activation [100]. In this field, nanotechnology has developed biomimetic anticancer vaccines that range from cell-derived nanoparticles, virus-like particles, artificial antigen-presenting cells, cell membrane-coated nanoparticles, PD-1 nanovesicles, IDO1 inhibitors, and engineered megakaryocytes that render platelet nanoparticles expressing programmed death ligand 1 (PD-L1), functionalized platelets carrying anti-PD-L1 antibodies or NK cell membrane-coated nanoparticles that lead to immunogenic cell death in tumor cells [101]. Engineered macrophages are capable of targeting and neutralizing solid tumors [102].

Moreover, vaccines can be made from whole tumor cell lysates and blood-circulating monocytes, which can be differentiated ex vivo into monocyte-derived dendritic cells (moDCs), primed with whole tumor lysates (binding to neoantigens and presenting them loaded in HLA-I receptors), and then reinfused into the patient, eliciting an anti-tumor response [97,103,104,105,106].

Furthermore, DC vaccines have been used to treat melanoma and glioblastoma. Peptide vaccines are being evaluated with granulocyte-macrophage colony-stimulating factor (GM-CSF) for breast cancer. Multiple peptides are under study for melanoma, and whole tumor cell lysates are being researched for pancreatic cancer, non-small cell lung cancer, melanoma, and colorectal cancer [107]. Targeting the TME and tumor-associated cells is an emerging, promising approach, which we discuss below.

This field is experiencing a rapid expansion, with great progress in RNA-based tumor vaccine clinical trials for NCSLC [108], as well as mRNA-4157 for significantly reducing melanoma risks [109], and promising results for DNA- or peptide-based HPV vaccines in the context of cervical cancer [110]. Another interesting and novel approach to triggering the immune system’s anti-tumoral potential is using bacterialized tumor cells as a vaccine in murine models, which facilitate DC migration and alter the TME within the draining lymph nodes [111].

DCs have immense potential because their extensive peptide-presentation capabilities enable T-cell priming, as they are a natural bridge between innate and adaptive immune responses. Tumor-DC-based vaccines enhance T-cell activation and TME infiltration, favoring anti-tumoral capabilities [100].

## 13. Checkpoint Inhibitors, CAR-T/NK Cells, and Genetically Engineered Macrophages (CAR-Mφ)

Checkpoint inhibitors are used to prevent tumor- and TME-induced suppression of T-cell proliferation. Anti-tumor immune responses begin when APCs (DCs, human macrophages, or B-cells) engulf tumor-associated antigens (TAAs) or whole tumor cells. This likely occurs in the TME during the early stages of its establishment, and these cells migrate to secondary lymphoid organs, where they present TAAs on their HLA molecules. Depending on the antigen-processing and presentation pathway, peptides are loaded onto HLA-I or HLA-II molecules, triggering the activation and proliferation of CD8^+^ or/and CD4^+^ lymphocytes if their TCRs specifically recognize the antigen. Cytotoxic T-cells exit the secondary lymphoid organs via the lymphoid circulatory system and exert their anti-tumor function by secreting granzymes and perforins or by triggering FasL-mediated apoptosis [16,112].

T-cell activation requires three signals for full activation: the first signal is delivered through TCR interaction by TCR variable αβ receptor domain-peptide interactions, that are further stabilized by either CD4 co-receptor for T-helper cells or CD8 for cytotoxic T-cell mediated TCR interactions, the second necessary signal is provided by co-stimulatory interactions, classically CD28 in the T-cell, which binds to B7.1 or B7.2 in the APC as well as a third soluble signal that normally implies cytokine crosstalk and further integrin dynamic associations such as LFA-1-ICAM-I as well as CD2-LFA-3 in T-cell-APC stabilize immune synapse and render fully activated cells that can proliferate in secondary-lymphoid tissues. CTLA-4 expression competes with CD28 on the cell surface, thereby inhibiting T-cell proliferation. PD-L1 engagement of PD-1 inhibits TCR and co-stimulatory signaling, thereby reducing T-cell activation and effector function and promoting a dysfunctional/exhausted phenotype that activates phosphatases that inhibit the propagation of activating ITAM-dependent signaling. PD-L1 is overexpressed in tumor cells and tumor-associated cells, generating an immunosuppressive milieu supported by regulatory T-cells (Tregs), and indolamine 2,3-dioxygenase 1 (IDO-1) favors immune suppression. This knowledge has opened new paths in the fight against cancer, as monoclonal antibodies against PD-L1 and CTLA-4 are clinically used to prevent T-cell anergy or cell death, thereby boosting anti-tumor adaptive immunity and showing promising results [112].

Tumor neoantigens have opened a path to designing engineered immune cells that express a receptor for the neoantigen, directing them specifically to the tumor microenvironment and enabling them to target either tumor cells or tumor-supporting cells, thereby boosting anti-tumor capabilities of the host immune system and helping eliminate the tumor. This group is currently expanding, and T-cells, NK cells, DCs, and macrophages are the principal engineered immune cells generated [113].

Chimeric antigen receptor-T (CAR-T) cell technology is an expanding field in immunotherapy. CAR-T cells are T-cells genetically engineered to express antigen-specific receptors not restricted by T-cell HLA molecules. These receptors are chimeric, composed of an antibody-derived single-chain variable fragment fused to a transmembrane domain and an intracellular signaling domain from the TCR/CD3 complex. Several techniques are applied to express these receptors, ranging from plasmid or viral vectors to electroporation [114].

CAR-T technology is extensively applied to hematopoietic tumors and has become a hotspot in oncological immunotherapy [115,116,117,118]. However, CAR-T therapy is limited in solid tumors by antigen heterogeneity, impaired trafficking and the immunosuppressive TME, while its clinical implementation is additionally constrained by treatment-related toxicities, particularly CRS, ICANS, hematological toxicity and infectious complications. Graft-versus-host disease (GvHD) is not a characteristic toxicity of conventional autologous CAR-T products, although it remains an important consideration for allogeneic T-cell platforms, whereas CAR-T exhaustion primarily represents a limitation of persistence and therapeutic efficacy rather than an adverse event. Together with manufacturing and logistical constraints, these considerations have driven the development of CAR-NK cells as an alternative immune-effector platform with a distinct, emerging safety profile [119,120]. This is further discussed in the next section of the review.

## 14. CAR-Macrophages and Other Genetically Modified Macrophages

The concept of activating macrophages as a cancer therapy is not new. In the 1980s, researchers generated ex vivo-activated macrophage killer cells (MAKs) by isolating monocytes through leukapheresis, subsequently differentiating them into macrophages in vitro, polarizing them with IFN-γ to a proinflammatory state, and then reinfusing them into cancer patients in an autologous manner [121]. This example illustrates that favoring endogenous macrophage activation, as occurs during adaptive immune activation (BCG, etc.), has long been considered a potential cancer therapy. In this context, many different approaches have been developed and are being continuously tested in clinical trials. Building on previous strategies, CAR-macrophages or genetically engineered macrophages appear to be a novel approach to tackle solid tumors [122,123], particularly in those where CAR-T cell therapies have not been as successful as expected.

CAR-macrophages rely on phagocytosis and antigen presentation to exert their anti-tumor activity, and they have been shown to synergize with anti-PD-L1 and anti-CTLA-4 immune checkpoint inhibition [124]. These professional phagocytic cells can produce and release proinflammatory cytokines (IL-6, IL-12, IL-23, TNF-α, and IFN-γ), which orchestrate a coordinated response. By presenting tumor peptides loaded on their HLA-I and HLA-II receptors, human macrophages elicit either CD3^+^CD8^+^ or CD3^+^CD4^+^ activation, respectively (although cross-presentation is less frequent), and trigger complement activation and boost antigen-dependent cellular cytotoxicity (ADCC) in NK cells, driving IFN-γ-dependent NK activation that exacerbates Th1 activation, favoring an IFN-γ-positive feedback loop and IL-2 production. This, in turn, helps reverse the immunosuppressive milieu the tumor creates within the TME.

**Figure 4:**
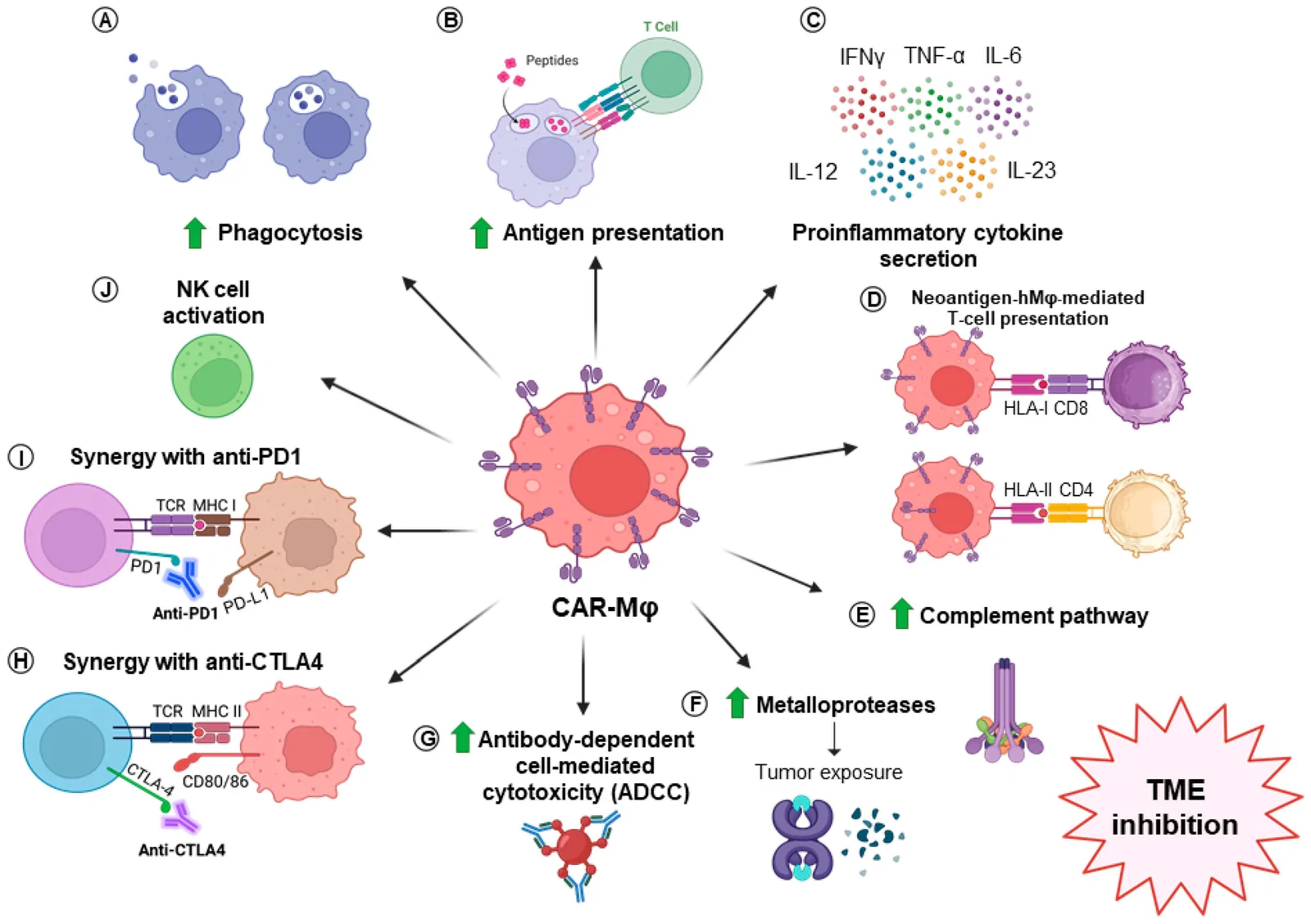
CAR-macrophages (Mφ) functional activation capabilities enhancement rendering anti-tumoral properties. A schematic functional macrophage anti-tumoral-related function enhancement is shown. (**A**) Phagocytosis, (**B**) antigen presentation, (**C**) pro-inflammatory cytokine production and secretion, (**D**) neoantigen-human macrophage-mediated T-cell presentation, (**E**) complement activation pathways, (**F**) enhanced metalloprotease secretion, controlled ECM remodeling/degradation to facilitate immune-cell infiltration (**G**) ADCC cytotoxicity, (**H**) synergistic approaches with anti-CTLA-4 or (**I**) PD-L1, immunotherapies and (**J**) NK cell activation. This figure was created in BioRender. https://BioRender.com/jcr0gpe (accessed on 23 July 2026).

Other molecular mechanisms once thought to be exclusively pro-tumoral, such as metalloproteinase (MMP) production, can be modulated to expose the tumor and allow immune cell infiltration. These mechanisms support a broader systemic response that reshapes the TME; however, they also create a pressing need to fine-tune the immune response to counter the tumor’s ability to adapt to dynamic changes through immunoediting and to identify novel tumor neoantigens to direct and confine the response exclusively to the tumor lesion. These broader immunomodulatory effects make control of inflammatory signaling and tumor specificity important design considerations. However, the clinical toxicity profile of CAR-macrophages remains incompletely defined and should not be extrapolated directly from CAR-T experience [124,125]. Figure 4 summarizes this information.

A trend explores human tumor-associated macrophage (TAM) reeducation of tropic TAM toward a proinflammatory phenotype [126]. Briefly, CAR-macrophage technology development is an expanding field in which additional modules are incorporated to enhance macrophage immune surveillance and tumor-restricting or killing capabilities. We propose a similar classification, although it is not standardized in the field; it is, to some extent, analogous to the standardized CAR-T classification by generations. The first generation featured a single-chain fragment variable (scFv), a hinge, and a single activation domain, aiming to boost phagocytic function. Receptors such as CD19 and CD22 fall into this category. These were later expanded with an additional module that increased T-cell activity. The first two generations used lentiviral or adenoviral vectors, whereas the third generation added a module that activates cytotoxic lymphocytes and stimulates antigen- and proinflammatory-dependent macrophage activation (e.g., anti-ALK), adding another regulatory layer to coordinate innate and adaptive responses. This third-generation approach aims not only to kill the tumor but also to target supporting stromal cells, leveraging non-viral delivery methods [127]. Additional approaches in CT26-HER2 murine models using circular RNAs encoding CARs have recently been developed [128].

Ovarian cancer has been a target for CAR-T and there are currently several clinical trials in recruiting phases, but due to the limited rate of success of CAR-T within the plethora of solid tumors, CAR-NK and CAR-macrophages are also being explored as a therapeutic venue aiming at HER2 and mesothelin in ovarian and breast cancer [127,129,130].

Generating CAR-CD47/HER2 macrophages could elicit a cytotoxic lymphocyte response and orchestrate Th1-dependent IL-2, IFN-γ and TNF-α release, promoting lymphocyte proliferation and tumor clearance. This rationale has now been evaluated in the first-in-human phase 1 trial of CT-0508 (NCT04660929), in which 14 patients with HER2-overexpressing advanced solid tumors received autologous anti-HER2 CAR macrophages without preceding lymphodepletion. No dose-limiting toxicities, severe CRS (grade ≥ 3), or ICANS were observed. These results support the preliminary tolerability of this approach, although the small cohort size precludes definitive conclusions regarding uncommon, delayed, organ-specific, or on-target/off-tumor toxicities. A separate initial clinical report of autologous mesothelin-targeted CAR macrophages in two patients with ovarian cancer also described a favorable early safety profile. Therefore, substantially larger prospective cohorts and longer follow-up will be required before the safety of CAR-Mφ can be reliably compared with CAR-T or CAR-NK platforms [125]. Moreover, IL-15 iPSC-derived CAR macrophages exhibited increased phagocytosis and HLA-I antigen-presenting receptors after targeting pancreatic and prostate cancers. This elicited a cytotoxic T-cell-dependent response, further enhanced by IL-15 secretion to recruit NK cells [130].

Beyond the novel therapeutic design targeting CAR-recognized molecular targets, other technical challenges are not directly related to CAR design constructs or their co-stimulatory domains but concern manufacturing and standardization processes. The ontological origin of macrophages [121,131,132] (human vs. non-human) and, within human macrophages, sources from less desirable to more suitable: THP1 cells, human peritoneal monocytes, umbilical cord stem cells, iPSCs and primary autologous human monocytes from peripheral blood mononuclear cell isolation. Their advantages, disadvantages, and clinical consequences of usage [132], as well as their elevated costs, must be considered.

Autoimmune and fibrotic-related diseases are other promising targets for CAR technologies [133,134], although these are beyond the scope of this review.

There are other genetically modified macrophage approaches taking advantage of CRISPR-Cas9 technology for SIRPA knockout in macrophages, or other strategies blocking the SIRP-α-CD47 axis, desensitizing macrophages to phagocytosis inhibition “do-not-eat-me signals”. IFN-γ overexpression or IL-2 production to boost NK and T-cell tumor cytotoxicity; engineered macrophages to produce and secrete bispecific T-cell engagers and express molecules that block angiogenesis and metastasis, such as NK-4; and macrophages coupled or modified for therapeutic drug delivery121. Hence, minimizing toxicity and side effects is the goal of precision medicine in immunotherapy. It is a rapidly developing technology, and due to limited clinical information, it is expected that there will be substantial improvements as well as novel identification of issues, but this approach is promising from a therapeutic perspective.

## 15. CAR-T Cell Therapy Progression

CAR-T cell therapy represents a transformative innovation in cancer immunotherapy, leveraging genetic engineering to redirect a patient’s T-cells to target and destroy tumor cells. This approach involves modifying T-cells to express chimeric receptors (CARs) that combine the antigen specificity of antibodies with the cytotoxic capabilities of T-cells [135]. Since its approval for the treatment of hematological malignancies such as B-cell ALL and large B-cell lymphoma, CAR-T therapy has shown unparalleled success in achieving remission in patients resistant to traditional therapies [69].

**Figure 5:**
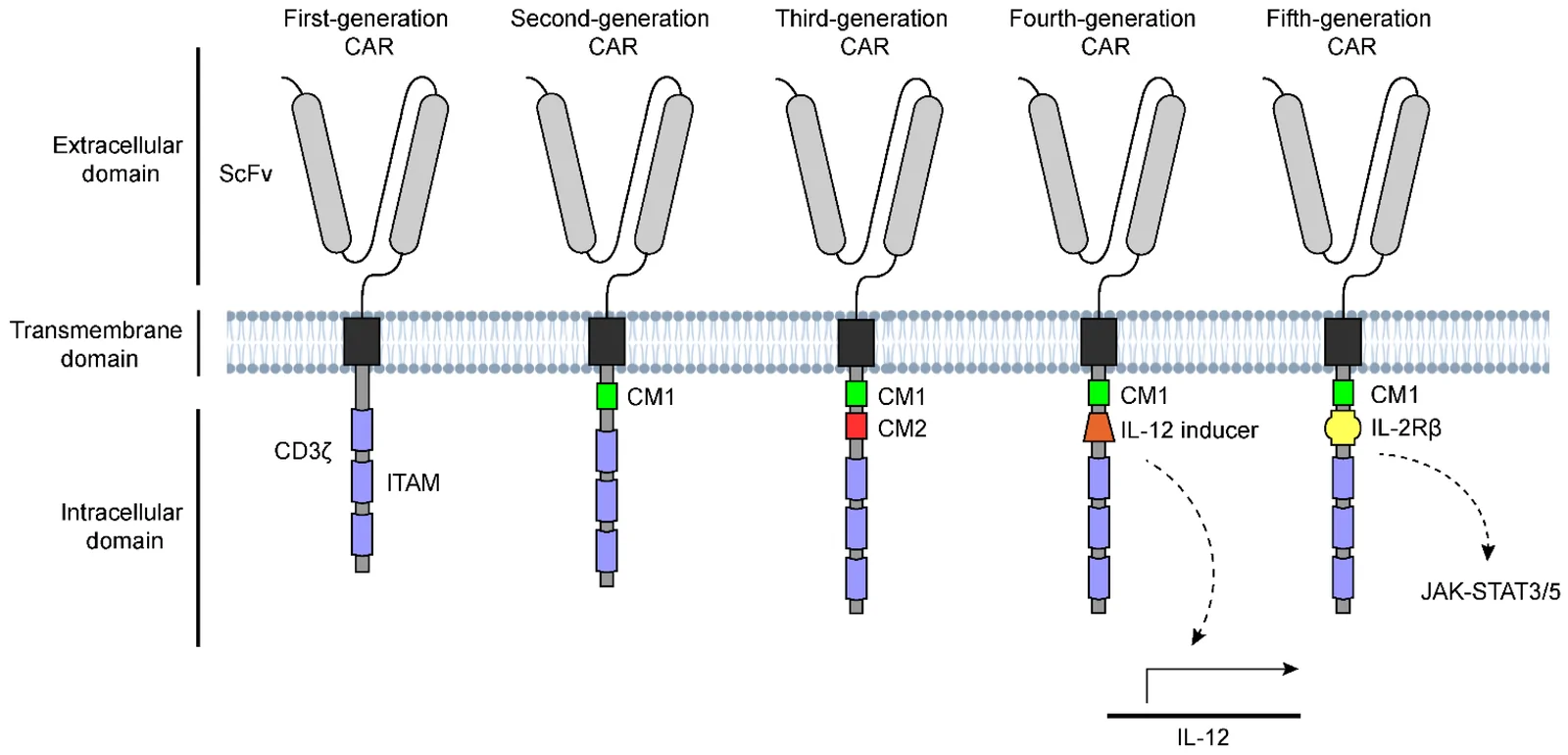
Evolution of the different CAR-T cell generations. The first generation includes a single-chain variable fragment (scFv) and intracellular signaling modules (CD3ζ or FcεRIγ) containing ITAM motifs. The second generation incorporates a costimulatory module (CM1), such as CD28, to enhance signal transduction. The third generation builds on this by adding a second costimulatory module (CM2) to the signal-transduction region, such as CD134 or CD137. Fourth-generation CAR-T cells add an inducible transgenic cytokine cassette (e.g., IL-12) to a second-generation CAR backbone. Finally, fifth-generation CAR-T cells engineer synergistic activation of triple signals from CD3ζ, CMs, and cytokine-inducing JAK-STAT3/5 pathways.

CAR contains three key domains: (1) an extracellular domain, consisting of the antigen recognition moiety and the hinge regions, (2) a transmembrane domain, and (3) an intracellular signaling module. These elements orchestrate antigen recognition and activation of T-cells, ultimately inducing cytotoxicity [136]. CAR-T cells have been developed over several generations, each improving persistence, safety, and efficacy. The primary driver of CAR evolution is enhancing the biological and signaling properties of the intracellular domain. For instance, second-generation CARs incorporate co-stimulatory domains (e.g., CD28 or 4-1BB), whereas third- and fourth-generation CARs include additional pathways to enhance activation, resistance to immunosuppression, or cytokine production [117,137] (Figure 5).

While the success of CAR-T therapy is well-documented in hematological cancers, several challenges remain untackled [116]. Tumor escape through antigen loss induced by tumor immunoediting [138], toxicities such as CRS and immune effector cell-associated neurotoxicity syndrome (ICANS), and the limited efficacy of CAR-T therapy in solid tumors, as well as the rapid CAR-T exhaustion, have prompted significant research into new targets and mechanisms [139]. Emerging strategies have been developed to focus on engineering more precise CAR-T cells to overcome immunosuppressive tumor microenvironments [136].

Safety is a central determinant of CAR-T clinical implementation. The two best-characterized acute toxicities are CRS and ICANS, although their incidence and severity vary substantially according to the CAR product, disease context, tumor burden, conditioning regimen and toxicity-grading system. Representative pivotal studies illustrate this variability. In ZUMA-1, axicabtagene ciloleucel was associated with grade ≥ 3 CRS in 13% of treated patients and grade ≥ 3 neurological events in 28%, whereas in the JULIET study of tisagenlecleucel, grade 3–4 CRS and neurological events were reported in 22% and 12% of patients, respectively; prolonged cytopenias and grade 3–4 infections were also observed in 32% and 20% of patients. These rates should not be interpreted as direct product comparisons because the studies differed in patient populations and toxicity-grading systems. CRS is an early systemic inflammatory syndrome characterized initially by fever and, as it worsens, hypotension and/or hypoxia. ICANS encompasses a distinct spectrum of neurological manifestations, including impaired attention and language, encephalopathy, tremor and seizures, with severe cerebral edema occurring rarely. Management is severity-adapted: IL-6 receptor blockade with tocilizumab is central to the treatment of clinically significant CRS, with corticosteroids incorporated for severe, persistent, or insufficiently responsive cases. In contrast, corticosteroids are the primary pharmacological treatment for clinically significant ICANS; tocilizumab is not recommended for isolated ICANS without concurrent CRS.

Toxicity surveillance should also extend beyond CRS and ICANS. Immune effector cell-associated hematotoxicity (ICAHT) is a frequent complication of CAR-T therapy and can manifest as profound, prolonged or recurrent cytopenias, thereby increasing the risk of infection and bleeding. Current consensus recommendations include exclusion of alternative causes of cytopenia, growth-factor support, transfusion and risk-adapted antimicrobial prophylaxis, with autologous hematopoietic stem-cell boost considered in selected severe and refractory cases. Infectious risk reflects the combined effects of lymphodepleting chemotherapy, prolonged neutropenia and lymphopenia, corticosteroid or other immunosuppressive treatment of immune-related toxicities and, depending on the CAR target, prolonged B-cell or plasma-cell depletion. A less common but potentially life-threatening hyperinflammatory toxicity is immune effector cell-associated hemophagocytic lymphohistiocytosis-like syndrome (IEC-HS), which can present with worsening cytopenias, hyperferritinemia, coagulopathy/hypofibrinogenemia and hepatic dysfunction and can develop as CRS is resolving. Recent consensus classifications additionally recognize non-ICANS neurological syndromes and on-target/off-tumor injury as clinically relevant immune-effector-cell toxicities, emphasizing that safety assessment must extend from the immediate post-infusion period into long-term follow-up [140,141,142].

CAR-T cell therapy has undergone significant advances since its inception in the late 1980s, progressing through several generations [143]. Each generation of CARs represents an effort to address specific challenges, such as safety, persistence, and efficacy [136]. The evolution of CARs is mainly driven by changes in the number and nature of their intracellular domains (Figure 5). Each generation is analyzed in detail, highlighting its structural and functional innovations.

***CAR-T first generation*****:** First-generation CARs were developed featuring a single intracellular signaling domain derived from CD3ζ or FcεRIγ. These CARs provided primary activation signals through immunoreceptor tyrosine-based activation motifs (ITAMs), mimicking natural TCR signaling pathways [63]. However, although they can target tumor antigens independently of HLA presentation, they do not provide the co-stimulatory signals needed for robust IL-2 production. Thus, their limitations, including poor T-cell expansion, limited cytokine production, and rapid exhaustion, became evident. These CARs required supplementation with exogenous IL-2 to sustain T-cell activity, thereby limiting their clinical utility [64].

***CAR-T second generation*****:** Second-generation CARs introduced a major innovation: the addition of co-stimulatory domains, such as those contained in CD28, 4-1BB (CD137) or OX-40 (CD134), to the intracellular signaling region [65,66]. This advancement allowed CAR-T cells to receive dual activation signals, and in general, the results were better for T-cell proliferation, persistence, and effector function than those observed in the first generation [144]. Specifically, CD28-based CARs are associated with rapid expansion and strong effector responses, while 4-1BB domains promote prolonged persistence and memory formation [141]. These modifications led to the development of the first Food and Drug Administration (FDA)-approved CAR-T therapies, such as tisagenlecleucel (Kymriah) and axicabtagene ciloleucel (Yescarta), for the treatment of CD19-positive hematological malignancies [69,140,145].

***CAR-T third generation*****:** Third-generation CARs were further improved by incorporating multiple co-stimulatory domains, such as those derived from CD28, 4-1BB, and OX40. These designs sought to amplify cytokine production, enhance anti-tumor cytotoxicity, and increase persistence [146]. For example, combinations such as CD3ζ-CD28-4-1BB showed superior in vivo expansion, tumor clearance and persistence in preclinical models [117,147]. However, the clinical benefits of third-generation CARs compared to second-generation constructs have been inconsistent. Although they offer theoretical advantages, increased signaling intensity has been associated with faster T-cell exhaustion and higher risks of toxicity, including CRS [72]. This has limited their widespread clinical applications to date. Moreover, second- and third-generation CARs face challenges in treating solid tumors because the TME is immunosuppressive.

***CAR-T fourth generation*****:** Fourth-generation CARs, also called T-cell redirected for universal cytokine killing (TRUCKs), aim to overcome the immunosuppressive effects of the TME. These CARs incorporate inducible cytokine cassettes, such as IL-12, which reprogram the TME to enhance anti-tumor responses. By releasing cytokines in a tumor-localized manner, TRUCKs activate innate immune cells, including macrophages and NK cells, thereby promoting broader effects and enabling the elimination of heterogeneous tumor cells. This strategy has been especially effective in preclinical models of solid tumors and has shown improved tumor clearance without the need for preconditioning regimens [67,148]. Interestingly, CAR-T cells with IL-12 release capability became less sensitive to suppression by Tregs and demonstrated enhanced cytotoxic activity, prolonged persistence, and elevated systemic IFN-γ levels in ovarian cancer mouse models [149]. However, constitutive IL-12 release carries the risk of supra-physiological IFN-γ levels, which can lead to uncontrolled over-activation of NK cells and macrophages. This strongly supports the rationale for expressing IL-12 under CAR signaling control to ensure localized release in the target tissue for at least these two critical reasons [150].

***CAR-T fifth generation*****:** Fifth-generation CARs represent the cutting edge of CAR-T design, incorporating additional signaling pathways to improve safety, efficacy, and control. These CARs often integrate the JAK/STAT signaling pathway by adding IL-2 receptor beta (IL-2Rβ), enabling triple-signaling activation (CD3ζ, co-stimulation, and cytokine-mediated signaling) upon antigen engagement [70]. Pharmacologically controllable or suicide switches can also be incorporated into CAR constructs to attenuate or terminate cellular activity in the event of severe toxicity. Importantly, these safety modules are not defining features of fifth-generation CARs and can, in principle, be incorporated across different CAR architectures. This precision reduces the risk of adverse events, such as off-target toxicity or excessive immune activation [72]. Although fifth-generation CARs are still in development, they hold promise for improving efficacy in solid tumors, where previous generations have faced significant challenges [117].

Common co-stimulatory modules CM1/CM2 are incorporated into second- and third-generation CARs, including CD28, 4-1BB, ICOS, OX40, and CD27, whereas additional domains such as Toll-like receptor TLR2, CD40, MyD88/CD40, GITR, DAP10, HVEM, and TLR4 have been explored in experimental CAR-T designs [151]. Table 1 and Table 2 summarize CAR-T and CAR-NK CM’s.

## 16. CAR-T Therapy Targeting CD19 and CD22

CD19-targeting CAR-T therapy has revolutionized the treatment of B-cell malignancies. Tisagenlecleucel (Kymriah) and axicabtagene ciloleucel (Yescarta), the first two FDA-approved and commercialized CAR therapies, target CD19 and have achieved promising remission rates in clinical trials for relapsed/refractory B-cell ALL and diffuse large B-cell lymphoma [140,145].

However, resistance mechanisms such as antigen loss or mutation allow tumor cells to evade single-target therapies. Dual-targeting CAR-T cells are designed to simultaneously target CD19 and CD22, improving efficacy and durability while reducing relapse rates in patients with refractory or relapsed disease [116,163]. Preclinical and clinical studies have shown promising results for bicistronic or tandem dual-target CAR-T therapies. Bicistronic CAR-T cells incorporating CD19/CD22 scFvs have been assayed in immune synapse experiments and have shown results similar to those in Jurkat T-cell in vitro models [164]. This tandem has demonstrated safety and efficacy in pediatric and young adult patients with B-cell ALL [135,165,166]. Additionally, tri-specific CAR-T constructs targeting CD19, CD20, and CD22 further enhanced tumor elimination by expanding coverage across B-cell markers [139]. Despite their success, dual-target CAR-T therapies still face challenges, including reducing or preventing off-target toxicity while maintaining efficacy. Innovative constructs are being developed, such as those utilizing humanized scFvs or incorporating cytokine signaling enhancements to improve therapeutic safety and the durability and potency of responses [116,167].

An additional target-dependent safety consideration for CARs directed against B-lineage antigens, particularly CD19, is prolonged B-cell aplasia and consequent hypogammaglobulinemia, which can increase the risk of late infections. Therefore, monitor serum immunoglobulin levels after therapy, and individualize immunoglobulin replacement based on the degree and persistence of humoral immunodeficiency and the occurrence of recurrent or severe infections [168].

## 17. CAR-T Therapy Using NKG2D Receptors

NKG2D (natural killer Group 2, member D) CAR-T therapy is an innovative approach that exploits the NKG2D receptor, a natural activating receptor expressed on NK cells, subsets of γδ T-cells and naïve human CD8^+^ cytotoxic T-cells [169]. Unlike traditional CAR-T therapies, NKG2D CARs are designed to recognize a wide array of stress-induced ligands (e.g., MICA, MICB, and ULBPs) expressed on the surface of a broader range of tumor cells [170]. This broad-spectrum targeting offers distinct advantages for combating tumor heterogeneity and resistance mechanisms [171,172].

Recent advancements have demonstrated the versatility of NKG2D CAR-T cells in the treatment of various cancers [71]. For example, IL21-NKG2D CAR-T cells have shown enhanced persistence and efficacy in treating multiple myeloma [173]. Similarly, CYAD-01, the first-generation autologous NKG2D CAR-T product, has proven effective in preclinical and early clinical trials [174]. In hepatocellular carcinoma, non-viral engineering methods have been employed to generate NKG2D CAR-T cells, offering a scalable and cost-effective approach [175].

Despite their potential, NKG2D CAR-T cells face challenges, including immunosuppressive tumor environments and fratricide risks arising from the expression of NKG2D ligands on T-cells [176]. Strategies such as cytokine support, nanotechnology, and improved engineering techniques are under active investigation to address these limitations [174,177,178]. With further research, NKG2D CAR-T therapies hold significant promise for expanding the application of CAR-T technology to diverse cancer types.

This is outside the scope of this review; however, for a more detailed, classified, and comprehensive analysis of the different specific types of CARs, please refer to these excellent reviews, which include: a table summarizing the various CAR-T therapies with clinical efficacy evidence [143] and a table listing the ongoing clinical trials utilizing both CAR-T and CAR-NK therapies against solid tumors [179]. Currently, there are clinical trials for colorectal cancer against CEA with a CD28 costimulatory domain, ROR-1 in breast cancer with a 4-1BB costimulatory domain, in gliomas against EGFvRIII, HER2, IL13Rα2 or B7-H3, hepatocellular carcinoma (GPC3^+^ or CD133^+^) as well as mesothelioma, ovarian and prostate, and pancreatic cancer with the aforementioned costimulatory domains in phase I or II [180,181]. These rapidly evolving technologies, which are continuously being assessed, will self-correct and identify novel issues and limitations to overcome, as the scientific community expects.

It is worth mentioning that immune cell activation requires both the proper signaling pathways as well as the metabolic rewiring that occurs simultaneously, which is essential for the activation process, and it normally implies central metabolic pathways such as glycolysis, the Krebs cycle, and the pentose phosphate pathway, as well as lipid and protein handling, as it is explained in the subsequent sections of the review.

## 18. Mitochondrial Metabolic Reprogramming in CAR-T Cells: Strategies for Enhanced Clinical Efficacy

The metabolic profile of CAR-T cells is crucial for their functionality, persistence, and anti-tumor efficacy. There is evidence that patients with chronic lymphocytic leukemia who receive CD19 CAR-T cells with increased mitochondrial mass are more likely to achieve a complete response [182]. Recent studies have highlighted the importance of reprogramming mitochondrial metabolism to overcome challenges like T-cell exhaustion and improve clinical outcomes [183]. Within the TME, where CAR-T cells face immunosuppression and energy depletion, mitochondrial reprogramming strategies aim to sustain their activity and enhance fitness and persistence while avoiding exhaustion [173].

One approach focuses on enhancing oxidative phosphorylation (OXPHOS) and fatty acid oxidation (FAO) to boost CAR-T cell longevity and resilience. Several studies show that modulating regulators such as PGC-1α to improve mitochondrial biogenesis and OXPHOS rejuvenates exhausted T-cells, restoring their cytotoxic capabilities [184,185]. Finally, treatment of activated CD8^+^ T-cells with 4-1BB agonist antibodies increased mitochondrial fusion and biogenesis, improving adoptive T-cell therapy outcomes by increasing PGC-1α expression [186].

Furthermore, targeting dysfunctional mitochondria by enhancing mitochondrial fusion and reducing reactive oxygen species (ROS) can restore T-cell function. Another promising strategy involves optimizing the balance between glycolysis and OXPHOS [173]. Similarly, arginine regulates glycolysis and mitochondrial oxidation, and exogenous arginine induces a metabolic transition from glycolysis to OXPHOS. A low arginine microenvironment also impairs the proliferation and function of CAR-T cells [187,188].

These findings underscore the importance of integrating metabolic reprogramming into CAR-T cell engineering to overcome metabolic bottlenecks that limit their therapeutic potential. Enhancing mitochondrial mass and overall mitochondrial health, thereby improving mitochondrial respiration, can overcome many challenges faced by CAR-T cells, especially against solid tumors [189]. In the context of physiopathological T-cell exhaustion, CAR-macrophages emerge as a possible functional solution [130].

## 19. Immune Synapse in CAR-T Cells Versus TCR-Mediated Synapse

Understanding the commonalities and differences between TCR- and CAR-T-evoked immune synapses, signaling and activation is essential and transcends T-TCR and CAR-T recognition. The comparison between TCR and CAR-T receptors is a key question that has been extensively covered in the literature [190]. CAR-T renders independent HLA-I or HLA-II T-cell activation for CD8^+^ or CD4^+^ T-cells, respectively. In terms of targets, normal T-cells rely on variable αβ domains within TCR chains, where professional APCs (normally DCs) present processed peptides via HLA-I or HLA-II, whereas CAR-T cells rely on ScFvs inspired by BCR Fab. Efficacy is still being fine-tuned, and so is safety [68,191,192], because off-target effects are possible. This is related to differential immune synapse architecture and function, as discussed below.

The immune synapse (IS) plays a critical role in T-cell activation and cytotoxicity. In T-cells, the canonical IS is formed by interactions between the TCR and peptide-HLA (pHLA) complexes on the APC, creating a highly organized structure displaying bull’s-eye, concentric zones: a central supramolecular activation cluster (cSMAC) containing TCRs and signaling molecules with low F-actin density; a peripheral SMAC (pSMAC) enriched with adhesion molecules, such as LFA-1 and a distal SMAC (dSMAC), rich in F-actin [193]. In contrast, the IS in CAR-T cells is less organized and more dynamic. CAR-T synapses lack the classical concentric architecture and exhibit smaller and less stable SMACs. This leads to rapid but more transient signaling driven by CAR clustering [194], and eventually to CAR-T cell exhaustion.

While TCR-evoked synapses prioritize sustained signaling for immune regulation and memory formation, CAR synapses are optimized for rapid and potent cytotoxicity. However, the intensity of CAR-T signaling can lead to early exhaustion, thereby reducing therapeutic durability [195]. Studies suggest that CAR synapse quality, including stability and clustering, is directly correlated with clinical efficacy [196]. For instance, Machine Learning-based analyses ex vivo revealed that CAR-T cells forming high-quality synapses resembling TCR-evoked canonical IS showed better clinical outcomes [197].

To address these differences, researchers have designed CARs to mimic certain features of TCR-mediated synapses. For example, adapting CAR constructs to improve spatial organization and signaling modulation could enhance persistence without sacrificing potency [198]. Additionally, strategies such as altering co-stimulatory domains or tuning CAR signaling thresholds are being explored to balance rapid cytotoxicity and sustained function [196]. Recently, it has been shown that introducing a CD3ε module into CAR induces liquid-liquid phase separation and promotes IS maturation, displaying canonical IS formation, and persistent cytotoxicity, thereby exhibiting improved efficacy in tumor models [195].

In summary, CAR-T cell IS lacks the canonical, monofocal concentric F-actin structure observed in TCR-controlled IS. Instead, it displays multifocal F-actin redistribution [193,194]. Proximal and distal signaling pathways in CAR-T cells are induced and terminated more rapidly than in TCR-mediated responses. This rapid signaling correlates with faster lytic granule recruitment and detachment from target cells, resulting in a faster but less sustained cytotoxicity. Bridging these differences by incorporating lessons from natural TCR signaling into CAR designs represents a promising approach for improving CAR-T therapies [193].

## 20. CAR-NK Cell Therapy: A Novel Approach in Immunotherapy

Natural killer cells, defined by CD56 expression and the absence of CD3 (CD56^+^CD3^−^), play a key role in immune surveillance. They eliminate abnormal cells through direct cytotoxicity or antibody-dependent cellular cytotoxicity, by binding to the Fc fragment of immunoglobulins on target cells. CAR-NK cells are an emerging alternative to CAR-T cells in cancer immunotherapy, offering advantages over CAR-T in some respects. However, CAR-NK therapy has been less developed than CAR-T cell therapy, likely because NK cell receptor biology is more complex. The NK killing effect depends on the coordination and balance of multiple receptors on their membrane surface, mainly divided into activated receptors (ARs) and inhibitory receptors (IRs), which hinder the further development of clinical NK-based immunotherapy [199]. Among their potential advantages, NK cells do not require antigen presentation via HLA molecules and can be used in allogeneic settings with a low observed propensity for graft-versus-host disease (GvHD). In contrast, most established CAR-T products rely on autologous T-cells and therefore require patient-specific manufacturing, which can delay treatment and may limit feasibility in patients with insufficient or poor-quality T-cell collections. This property supports the development of “off-the-shelf” CAR-NK therapies using NK cell lines, umbilical cord blood-derived NK cells, or induced pluripotent stem cell-derived NK cells, which can potentially reduce the manufacturing delays associated with autologous CAR-T therapy [120,199,200]. Conversely, CAR-T therapies require patient-derived T-cells to mitigate the risk of alloreactivity, which can significantly delay treatment and exclude some patients due to insufficient T-cell numbers [200]. This unique property enables the development of “off-the-shelf” CAR-NK therapies using existing NK cell lines, NK cells from umbilical cord blood, or from induced pluripotent stem cells, which can be rapidly infused into patients without the delays associated with autologous CAR-T cell manufacturing [120,201].

Additionally, a clinical trial with HLA-mismatched CAR-NK cells has demonstrated that patients do not develop notable toxicities such as cytokine-rseleasing syndrome (CRS) or GvHD, highlighting the safety and feasibility of this approach in allogeneic settings [202].

Beyond their compatibility benefits, CAR-NK cells kill tumors in two ways: CAR-dependent cytotoxicity and innate NK receptor-mediated killing. Even when they are genetically engineered to express a CAR, NK cells retain their natural ability to recognize and destroy tumor cells through their own receptors. This dual targeting improves anti-tumor efficacy and reduces the risk of tumor evasion. This complementary CAR-dependent and innate receptor-mediated recognition may broaden tumor-cell recognition and partially mitigate antigen escape; however, the durability of CAR-NK responses remains limited by challenges including cellular persistence and trafficking [200].

A key potential advantage of CAR-NK therapy is its favorable safety profile to date in allogeneic settings, although clinical evidence remains substantially less mature than for CAR-T therapy. In a phase 1/2 study of cord blood-derived CAR19/IL-15 NK cells, 37 patients with CD19-positive B-cell malignancies were treated; none developed neurotoxicity or GvHD, and only one patient experienced grade 1 CRS. Reversible hematological toxicity occurred in all patients and was attributed to the lymphodepleting chemotherapy rather than to the CAR-NK product itself [202]. A subsequent systematic scoping review encompassing 150 patients across ten published CAR-NK studies found no reported grade ≥ 3 CRS, ICANS or GvHD.

However, all included studies were uncontrolled, only four enrolled more than three patients, and considerable heterogeneity existed in cell source, CAR design, manufacturing and dosing [203]. Therefore, the low observed incidence of severe inflammatory, neurological and alloreactive toxicities is encouraging but should not yet be interpreted as a class-wide guarantee of safety. Moreover, treatment-related complications arising from preparative lymphodepletion, particularly cytopenias, remain clinically relevant even when direct CAR-NK-mediated CRS or neurotoxicity is uncommon [202].

Advances in CAR-NK cell therapy have focused on improving persistence, metabolic fitness, and tumor infiltration. Strategies such as engineering NK cells to express chemokine receptors (e.g., CXCR4) have been explored to enhance the trafficking of effector cells to tumor sites [204]. Another critical area requiring deeper investigation is how CAR signaling, which incorporates CD3ζ-derived activation motifs originally adapted from T-cell physiology, influences NK cell responses. Unlike T-cells, where CD3ζ signaling aligns with their natural activation pathways, the impact on NK cell function is less understood [205]. As ongoing clinical trials continue to refine CAR-NK therapy, these strategies may contribute to improving the persistence, efficacy and clinical applicability of CAR-NK therapies for hematologic malignancies. A summary of CAR strategies is provided in Table 3. After examining single immunotherapies, it is important to note that the trend is shifting toward combination therapies because of tumor complexity.

## 21. Combinatorial Therapies Targeting the Tumor Microenvironment (TME): Both Tumor Cells and Tumor-Supporting Cells

The complexity of tumor biology shows that targeting tumor cells alone is insufficient, because the immune system also needs to be functionally reprogrammed to fight it. Immune suppressor cells play a crucial role in supporting tumor growth, progression, and metastasis. Recently, several cell types involved in creating a tumor-supportive microenvironment have been identified, including stromal support cells (CAFs and adipocytes) and various recruited immune cells, such as TAMs, TANs, TAPs, myeloid-derived suppressor cells (MDSCs), and T-regulatory cells (Tregs). Targeting these cells that help maintain and advance tumors can be a key strategy for promoting tumor clearance, reversing immune evasion, and developing effective, targeted therapies that can ultimately save lives.

## 22. Targeting Tumor-Associated Macrophages (TAMs)

Human macrophage activity is relevant in various inflammatory contexts such as cancer, where they may have a dual role [206]: in the early tumor stages, human macrophages recognize and prevent the growth and development of tumor cells [207], which is an essential property of immune surveillance. However, if the neoplastic lesion evades the immune system, the tumor microenvironment (tumor cells and stromal supporting cells) can recruit human macrophages and re-educate them to promote and maintain tumor progression [208,209,210,211]. These cells suppress the anti-tumor action exerted by lymphoid cells in a physiological context [212]. This re-education of the phenotype by human macrophages is usually referred to as tumor-associated macrophages (TAMs).

Virchow first described the link between cancer and inflammation in 1867 [56]. Since then, extensive knowledge has been generated and inflammation and immune system evasion have been recognized as two features of the Hallmarks of Cancer [213,214,215], as well as the existence of tumor-supporting cells that are either adjacent to the tumor (stromal cells, fibroblasts, angiogenic vascular cells, epithelial cells) or recruited from the bloodstream (innate and adaptive immune cells) [216,217].

TAM promote tumor progression by the production of VEGF, EGF, PDGF, IL-10, TNF-α, and TGF-β, which favor angiogenesis, invasion, and metastasis by secreting metalloproteinases into the extracellular matrix (ECM), such as MMP2-9, and immunosuppression by IL-4, IL-10, PGE_2_ [56,208,218,219,220,221,222,223,224].

Furthermore, TAM have been associated with poor prognosis in breast cancer [56,225,226], prostate [227,228], bladder cancer [229], endometrial carcinoma [230], squamous cell carcinoma, kidney cancer and follicular lymphoma, whereas in others, these cells have been linked to a good prognosis; this has been reported in gastric cancer [231,232], colorectal cancer [228,233] and melanoma [219]. The existence of TAM has also been reported in ovarian cancer [228,234], pancreatic cancer [235], hepatocellular carcinoma [236], lung cancer [228], brain tumors [237], sarcomas [238] and leukaemia [239].

There is controversy in the literature regarding TAM classification; some classify this phenotype as a fourth type of hM2, while others assert it is a proinflammatory-like phenotype. However, it has recently been identified as an intermediate-activated phenotype due to the co-expression of both hM1 and hM2 well-established markers [240]. The field is currently expanding, and TAMs are considered a dynamic cell type with several functional subsets depending on the tumor microenvironment location [222,240,241,242].

Functionally, TAM immunosuppression induces NK and CD3^+^CD8^+^ cell blockade, as well as HLA Class I and II downregulation (immunoediting). TAMs also inhibit dendritic cell maturation, and T-cell activation [219,240,243,244,245]. Other myeloid-derived cells play a role in immunosuppression, such as MDSCs or tumor-associated neutrophils (TANs) [208,244,246,247,248,249,250,251].

Metabolic regulation appears to drive modulation of the immune response. In the case of TAMs, the hypoxia generated at the necrotic core and neoangiogenic regions within the tumor favors HIF-1α translocation to the nucleus, and the enhanced glycolysis renders lactate, which is also produced by the tumor cells via the Warburg Effect, inducing anergy in NK and T-cells, which require glucose, glutamate, glutamine, serine and cysteine to be activated. However, because TAMs are more avid for these metabolites, lymphocytes cannot compete for these nutrients in this microenvironment and therefore cannot be activated to neutralize the tumor.

Moreover, end-products, such as lactate, have recently been recognized as additional epigenetic modifiers, as lactylation of lysine residues due to lactate accumulation seems to contribute to reprogramming metabolic functions and transcription in the context of cancer [252,253]. In addition, PD-L1 is upregulated by HIF-1α, which is expressed on the TAM cell surface. It binds to PD-1, triggering apoptosis or anergy in cytotoxic lymphocytes and inhibiting phagocytosis [254]. In addition, TAMs express IDO-1, which depletes tryptophan and increases kynurenine, favoring the recruitment of T regulatory cells to the TME and enhancing the immunosuppressive local environment [240,244,255,256]. Therefore, it is not surprising that TAMs and all immune-suppressive cell types have become targets for the development of precision medicine therapies.

There are four principal venues aiming at TAMs: inhibiting Mφ recruitment, TAM reprogramming into M1-like macrophages, blocking inhibitory signals affecting effector immune cells and depleting TAMs [123,246,257]. Inhibiting human macrophage recruitment by tumors prevents tumor growth and expansion. Several therapeutic strategies have focused on targeting the molecular axes that regulate monocyte infiltration. Both monoclonal antibodies and inhibitors have been developed for the CCR2-CCL2 axis, such as PF-04136309, with carlumab alone or in combination with FOLFIRINOX (oxaliplatin, irinotecan, leucovorin and fluorouracil) and CXCR4-CXCL12. Other approaches aim to induce metabolic switching; this is the case for the CSF1-CSF1R signaling axis (inhibitors BLZ945, monoclonal antibodies (mAbs): Lagnotuzumab for CSF1 and cabiravizumab and emactuzumab against the receptor). The third category is related to reactivating phagocytosis and abolishing the “do-not-eat-me” signals expressed by the tumor, such as the CD47-SIRPα axis. The last category is directed at TAM depletion; the most extended tactics are the following: anti-VEGF mAbs, liposomal clodronate, zoledronic acid, and trabectedin [123,221,238,240,244,245,255,258,259].

Combined therapies targeting TAM with other approaches offer a way to address cancer’s complexity and improve oncological treatment success. The objective is to aim specifically at TAM in combination with radiotherapy, chemotherapy, and immunotherapy (immune checkpoint blockade), or any of these permutations are being evaluated [260].

## 23. Neutrophil and Platelet Cooperation with Suppressive Myeloid Cells

Reprogramming TAMs requires accounting for the myeloid and platelet circuits that sustain their local states. Tumor-derived colony-stimulating factors can expand myeloid populations, whereas CXCR2 ligands recruit neutrophils [244]. TAN implies tumor association; polymorphonuclear MDSCs (PMN-MDSCs) and monocytic MDSCs (M-MDSCs) are pathologically activated suppressor populations in the context of tumoral biology; in physiology, they regulate the immune response. The identification of tumor-associated cells requires functional suppression assays alongside phenotype: CD66b expression or a suppressive transcriptional signature alone cannot establish PMN-MDSC identity [261]. TANs therefore overlap with, but are not interchangeable with, PMN-MDSCs; N1/N2 phenotypes do not capture this continuum [244,261]. ARG1-dependent arginine depletion and reactive oxygen/nitrogen species can attenuate T-cell responses, with their contributions varying between MDSC subsets and tissue sites [261,262].

Reciprocal signaling extends suppression beyond direct lymphocyte inhibition. In murine 4T1 tumors, contact-dependent MDSC–macrophage crosstalk increases MDSC-derived IL-10 and reduces macrophage IL-12 [263]. MDSCs can additionally seed the TAM compartment: transfer experiments in murine tumors show hypoxia/HIF-1α-dependent macrophage differentiation, establishing a conditional developmental link between suppressor populations [262].

In murine pancreatic ductal adenocarcinoma, tumor-derived IL-34 cooperates with neutrophil-derived PGE_2_ to establish CD138^+^ TAMs; their CXCL1 recruits neutrophils and SAA3 promotes a suppressive Siglec-F^+^ state that replenishes PGE_2_ [264]. Genetic and pharmacological perturbations support causality in mice; CD138^+^ TAM and neutrophil co-enrichment in patient tumors provide translational support without establishing the complete human circuit [264].

Tumor-associated platelets (TAPs) couple these networks to vascular dissemination. Tumor–platelet contact triggers platelet CXCL5/CXCL7 release, recruiting CXCR2^+^ granulocytes to early pulmonary metastatic niches in mice [265]. Platelet surface GARP (glycoprotein A repeats predominant) supports TGF-β activation; platelet-specific GARP deletion improves adoptive T-cell therapy and reduces systemic MDSCs in murine models, linking platelet activity to suppressor-cell accumulation without proving direct MDSC reprogramming [266]. Separately, tumor-induced neutrophil extracellular traps (NETs) can shield malignant cells from CD8^+^ T-cell and NK-cell contact [267]. These mechanisms explain how effector recruitment can coexist with ineffective target engagement.

The direction of these circuits is niche-dependent: neutrophil trogocytosis restrains suppressive macrophage states in murine pancreatic cancer liver metastases [268], whereas IL-36γ-armored CAR-T cells engage neutrophil-mediated tumor killing and endogenous T-cell responses in mouse models [269]. These findings make functional and spatial context essential when selecting myeloid interventions to complement CAR-Mφ, CAR-T and CAR-NK therapy.

## 24. Proteolytic and Metabolic Organization of the Tumor Microenvironment

CAF-organized ECM, perfusion and cellular metabolism jointly define oxygen and pH gradients. Hypoxia stabilizes HIF-1α and can favor glycolytic adaptation; proton-coupled lactate export and inadequate vascular clearance promote extracellular acidosis [217,240]. Neither glycolysis nor metabolite uptake maps directly onto a suppressive phenotype: preferential glucose uptake per cell by myeloid populations and glutamine uptake by malignant cells in experimental tumors reflect cell-intrinsic nutrient partitioning rather than universal TAM-driven lymphocyte starvation [270]. Macrophage metabolism can itself reshape these gradients: in murine tumors, REDD1 deficiency increases TAM glycolysis, curtails endothelial glucose use and normalizes vessels, reducing hypoxia and metastasis [271]. Here, competition favors endothelial cells, restraining tumor growth. The consequences for engineered effectors therefore depend on cellular state and location, extending the mitochondrial considerations discussed above.

These gradients shape macrophage instruction and substrate availability. Tumor-derived lactic acid induces HIF-1α-dependent VEGF and ARG1 expression in murine TAMs. This response also occurs under normoxic culture conditions, showing that HIF-dependent macrophage programs need not identify hypoxic niches [272]. Acidosis also engages proton-sensing GPR65: genetic perturbation in strongly acidic melanoma models reduces neutrophil-like infiltrates, while GPR65 inhibition suppresses acid-induced CXCL2/3/5/8 expression in human monocyte-derived macrophages [273]. These human-cell results support pH-sensitive chemokine regulation, not a universal TAM phenotype. Conversely, sodium lactate at controlled pH sustains TCF1^+^ CD8^+^ T-cell states and antitumor activity in experimental systems [274]. Lactate concentration, proton activity and oxygen availability therefore require separate interpretation.

Acidosis also changes the proteolytic landscape: cultured human melanoma cells at pH 6.8 increase MMP-2/-9 and cathepsin secretion [275]. This does not establish a universal acidic optimum for MMP catalysis; proteolysis depends on zymogen activation, tissue inhibitors of metalloproteinases (TIMPs) and substrate accessibility [276,277]. In experimental tumors, TANs and selected TAM states supply TIMP-1-free proMMP-9 [276], and MMP-9-mediated mobilization of matrix-bound VEGF links remodeling to vascular growth [278]. Vessel function then modifies oxygen delivery and acid clearance, closing a context-dependent feedback loop. Conversely, HER2-triggered CD147-based CAR-Mφ increase MMP expression, reduce collagen, and facilitate T-cell infiltration in murine breast tumors [277]. Proteolysis can therefore support either dissemination or therapeutic access. Spatially controlled matrix remodeling, coupled to metabolic fitness, offers a preclinical rationale for coordinating CAR-macrophages with lymphocyte therapies; clinical benefit remains unproven.

Vascular and matrix exclusion, immunosuppressive cellular networks, antigen heterogeneity, and metabolic stress make this TME milieu complex and affect physical parameters essential for immune regulation, such as pH, cellular steaminess, and cytokine and growth factor production, which in turn regulate oxygen and nutrient disposal, angiogenesis, metabolic rewiring, immune function, and ultimately patient prognosis.

## 25. Tumor-Infiltrating Lymphocytes (TILs)

As the primary effectors of adaptive anti-tumor immunity, tumor-infiltrating lymphocytes are a fraction of circulating T-cells that successfully penetrate the tumor bed and engage transformed cells [279]. Their density, phenotype, and spatial distribution are among the most robust predictors of clinical outcomes and responses to immunotherapy [280].

Tumor-infiltrating lymphocytes encompass diverse CD8^+^, CD4^+^ (including TFh-like and Treg) and γδ populations that settle within tumor parenchyma and adjacent immune niches. Single-cell and spatial studies have now resolved a hierarchical CD8^+^ landscape in which TCF1^+^PD-1^+^ “stem-like/progenitor-exhausted” cells maintain the proliferative potential that fuels effector pools under chronic antigen exposure, particularly within antigen-presenting niches and tertiary lymphoid structures (TLSs) [281,282].

Phenotypic markers that enrich lymphocyte tumor reactivity include CD39 and CD103, which are often co-expressed, and minimal transcriptional signatures distinguishing bona fide tumor-specific from bystander TILs have been identified across different tumor types, such as lung cancer or melanoma [283,284]. Importantly, TLS architecture, especially B-cell-rich, maturing TLSs, correlates with improved immune infiltration and response to checkpoint blockade, with spatial metrics emerging as predictive biomarkers [285].

Clinically, the FDA’s approval of Lifileucel (Amtagvi) for previously treated unresectable/metastatic melanoma, the first cellular therapy approved for a solid tumor [286], constitutes a milestone in the field. A phase II study reported an objective response rate of ~31% with durable complete responses; five-year analyses now demonstrate sustained benefit without novel long-term safety issues [287].

Ongoing efforts aim to generalize TIL therapy beyond melanoma via improved manufacturing (e.g., closed-system, tumor-reactive cell enrichment such as CD137^+^ selection; “young-TIL” protocols), rational combinations (checkpoint blockade, targeted agents), and optimized cytokine support (next-generation IL-2 agonists engineered to favor T effector signaling over Treg signaling). However, none have been approved yet [288]. Parallel biomarker studies integrate tumor-intrinsic features, TCR clonality, TIL state composition, and TLS metrics to refine patient selection [289] within the umbrella of personalized precision medicine.

## 26. Cancer-Associated Fibroblasts (CAFs)

Cancer-associated fibroblasts constitute the dominant stromal population within most solid tumors, where they orchestrate structural remodeling and paracrine signaling that profoundly shape immune infiltration. Initially, these populations were considered a uniform tumor-promoting cell type, but CAFs are now recognized as heterogeneous, plastic populations with context-dependent functions ranging from barrier formation to immune regulation [290], thereby modulating patients’ outcomes along with other microenvironment-related stromal cell types.

CAFs are a dominant, adaptable stromal cell lineage that influences extracellular matrix structure and biomechanics and, consequently, the immune landscape of the tumor microenvironment. scRNA-seq and spatial multi-omics reliably identify functional CAF subtypes across tumors: myofibroblastic CAFs (myCAFs; matrix/contractile, near the tumor), inflammatory CAFs (iCAFs; rich in cytokines/chemokines, located further), and antigen-presenting CAFs (apCAFs; HLA-II^+^, derived from mesothelial cells in some cases), along with recently identified metabolically specialized states [291]. These states coexist, interconvert, and form conserved spatial neighborhoods that influence T-cell infiltration and therapeutic response, highlighting that CAFs can be tumor-promoting, context-dependent, and occasionally tumor-restraining [292,293].

The relative abundance of CAF subtypes is essential. For instance, in pancreatic ductal adenocarcinoma, iCAFs (IL-6/LIF-high) and apCAFs (HLA-II^+^ with non-canonical antigen presentation) often promote immunosuppressive and immune-excluded environments, while specific fibroblast lineages, like CD105^−^ and collagen-rich/α-SMA^+^ stromal areas, can play containment roles, showing that indiscriminate stromal removal might unexpectedly worsen the disease [293].

Mechanistically, CAF programs orchestrate immune exclusion and immunotherapy resistance through TGF-β–driven stromal remodeling and chemokine barriers (e.g., CXCL12–CXCR4). Blocking TGF-β signaling in the stroma restores T-cell penetration and synergizes with PD-(L)1 blockade in preclinical/early translational studies [294]. Therapeutic strategies range from depletion (e.g., fibroblast activation protein (FAP)-directed) to reprogramming and payload delivery. For instance, FAP-targeted IL-2 variant simlukafusp alfa (FAP-IL2v) has shown tolerability and preliminary activity when combined with atezolizumab in phase I/II cohorts of squamous cancers [295]. In parallel, FAP-targeted PET (FAPI) has matured into a practical imaging biomarker of CAF burden with theragnostic potential; emerging data link high FAPI uptake to reduced anti-tumor immunity and worse immunotherapy outcomes, suggesting a role in patient selection and response monitoring [296]. Nonetheless, CAF targeting remains nuanced due to heterogeneity and the risk of on-target/off-tumor effects; therefore, the current consensus favors subtype-aware modulation over indiscriminate ablation.

## 27. Impact of Metabolic Reprogramming on Antitumor Therapies

Metabolic reprogramming is widely recognized as a hallmark of cancer and a critical determinant of immune cell function within the TME. Importantly, it may also influence antitumor therapy [297]. Recent breakthroughs emphasize that these metabolic alterations do not merely fuel autonomous tumor growth; rather, they establish intricate metabolic crosstalk among neoplastic cells, stromal compartments, and infiltrating immune populations [298].

This complex interplay of nutrient competition, metabolite signaling, and systemic metabolic fluctuations plays a decisive role in determining the efficacy of modern antitumor therapies, particularly immunotherapies. Understanding how specific metabolic pathways modulate immune evasion, therapeutic resistance, and cellular stemness is paramount to designing highly synergistic and effective clinical interventions. However, given the vast heterogeneity of metabolic and immunological networks across patient cohorts, predicting immunotherapy response requires sophisticated analytical frameworks.

Integrating numerous multimodal, patient-level biomarkers—including metabolic profiles, genomic alterations, and spatial immune landscapes—significantly improves the accuracy of immunotherapy response predictions [299]. Nevertheless, achieving robust generalizability across diverse clinical settings remains a substantial challenge, underscoring the need for standardized, scalable multimodal diagnostics. A few significant examples illustrate this metabolic contribution to improving oncotherapy:

***Macrophage glycolysis and immune evasion***: Macrophages are glycolytic cells that consume glucose and release large amounts of lactate [300]. Both metabolites help regulate immunometabolism in cancer. Reprogramming intracellular glucose metabolism tightly governs immune cell polarization. One regulatory point is sedoheptulose kinase (CARKL/SHPK, encoded by the SHPK gene), a crucial metabolic sensor required to reprogram activated macrophages [301,302]. Modulating CARKL expression alters glucose flux through the pentose phosphate pathway, directly influencing whether macrophages adopt a pro-inflammatory or anti-inflammatory/pro-tumoral phenotype. Indeed, T-cells also rely heavily on this pathway to determine their immune profile [303]. Manipulating this metabolic node is a viable strategy for reprogramming the TME immune landscape to favor antitumor responses.

Upper glycolysis not only supports tumor metabolism; at the end of macrophage glycolysis, lactate serves as a potent metabolic fuel, signaling molecule, and protein modifier within the TME. In aggressive malignancies such as glioblastoma, tumor-associated macrophages (TAMs) and glioblastoma stem-like cells (GSCs) engage in highly specialized metabolic crosstalk. GSCs use macrophage-derived lactate to enhance DNA repair, promote cellular stemness, and accelerate overall tumor kinetics [252]. Concurrently, this lactate-mediated interaction drives robust immune evasion, highlighting how myeloid-derived metabolites reshape cancer stem cell phenotypes, induce post-transcriptional modifications (lactylation), and shield cancer stem cells from immune surveillance [304].

***Systemic metabolic interventions and innate immune signaling*****:** It is well known that systemic metabolic interventions, such as intermittent fasting, profoundly affect immune cell longevity and memory [305]. Intermittent fasting induces β-hydroxybutyrate production, which depletes long-lived plasma cells by displacing them from their specialized survival niches. While this impairs humoral immunological memory, it highlights the high plasticity of immune cells in response to systemic metabolic shifts. At the cellular level, the interface between lipid homeostasis and innate immunity is further underscored by the cyclic GMP-AMP synthase (cGAS)–stimulator of interferon genes (STING) pathway [306]. The cGAS-STING DNA-sensing machinery dynamically exploits and regulates intracellular lipid metabolism, which may enhance innate immune recognition of tumors and provide new therapeutic interventions.

Similarly, nutrient scarcity often triggers a severe imbalance in antitumor immunity [307]. Under normal physiological conditions, the mechanistic target of rapamycin complex 1 (mTORC1) senses amino acid availability. However, in the nutrient-limited inflammatory TME, inflammatory cytokines can aberrantly activate mTORC1 signaling in cancer cells. This dysregulation drives cancer cells to hyper-consume surrounding nutrients, effectively starving tumor-infiltrating CD8^+^ T-cells of essential amino acids [308]. Under this dysfunctional amino acid availability, CD8^+^ T-cells undergo apoptosis, severely blunting the host’s adaptive antitumor immune response.

***Adipose tissue crosstalk and ferroptosis resistance*****:** A prominent mechanism of therapy resistance involves interactions between cancer cells and peritumoral adipose tissue (PAT). Recent findings establish a direct mechanistic link, forming a PAT-kynurenine-ferritinophagy axis that promotes ferroptosis resistance [309].

Adipose tissue within the peritumoral niche secretes kynurenine, which cancer cells actively take up and metabolize into 3-hydroxykynurenine. This metabolite binds directly to nuclear receptor coactivator 4 (NCOA4), thereby inhibiting NCOA4-mediated ferritinophagy. By suppressing degradation of ferritin (ferritin heavy/light chain), it severely restricts the intracellular pool of free labile iron required to catalyze ferroptosis. Crucially, pharmacological inhibition of the kynurenine pathway has been shown to synergize with anti-PD-1 immunotherapy, effectively overcoming resistance to ferroptosis and suppressing tumor progression in preclinical models [310].

## 28. Perspectives in CAR-T Dependent Therapies

The next phase of CAR-T–dependent therapies will likely be defined not solely by increased receptor complexity and function, but by the ability to control when and how these cells act. This means that spatiotemporal T-cell activation is pivotal. This approach aims to maximize their therapeutic potential while reducing collateral side effects. In solid tumors, antigen heterogeneity, poor cell trafficking and homing, and the suppressive tumor microenvironment remain major barriers. This suggests that effective CAR-T cells need to serve not only as cytotoxic effectors but also as local organizers of anti-tumor immunity [180]. Recent studies support this view: CAR-T cells engineered to deliver PD-L1-targeted IL-12 locally demonstrate how spatially restricted immunomodulation may enhance intratumoral activity while limiting systemic toxicity [311], whereas IL-36γ-armored CAR-T cells may reprogram the tumor microenvironment, engage neutrophils, and promote endogenous anti-tumor responses beyond the CAR-targeted antigen [269]. Together, these findings suggest that progress in solid cancers will rely more on restoring broader immune activation and tumor access than on increasing tumor destruction alone. Simultaneously, CAR-T therapy is expanding beyond oncology.

Accordingly, safety should increasingly be treated as an independent engineering axis, incorporating spatially restricted activation, refined antigen recognition and controllable CAR activity to reduce systemic inflammatory and on-target/off-tumor toxicities without compromising antitumor function.

The clinical activity of CD19-targeted CAR-T cells in certain severe autoimmune diseases supports the idea that engineered lymphocytes can induce significant immune resetting and serve as tools for targeted immune reprogramming in various disease settings [312]. However, this broader therapeutic scope also highlights the urgent need to make safety a central focus of the design. The role of CD4^+^ CAR-T cells in immune-related adverse events after B-cell maturation antigen (BCMA) CAR-T therapy emphasizes the importance of understanding product composition, expansion kinetics, and tissue-specific toxicities [313].

The issue with CAR-T does not exclusively lie in the first activation signal for the CAR. Antigen escape, mutations, tumor immunoediting, heterogeneity, and clonal selection can occur; therefore, there is a pressing need to develop multi-antigenic approaches. A physical barrier, such as the extracellular matrix, or a biochemically immunosuppressive milieu can prevent or inhibit T-cell activation, even when the neoantigen and the ScFv interact productively. This category includes CTLA-4 and PD-L1, which can be soluble or present on Tregs and APCs, respectively. The future of these immunotherapies will depend on how the scientific community solves the current issues. CAR-T therapies have shown efficacy in liquid tumors because, by nature, they are not confined by the extracellular matrix; therefore, no physical barrier prevents T-cells from recognizing and specifically binding to the tumor. Overcoming ECM barriers is one of the greatest challenges, but there are others, including neutralizing inhibitory signals for T-cell activation (using anti-PD-L1 or anti-CTLA-4) [314,315,316]. Although it is out of the central scope of this review, it is relevant to mention that epigenetics, metabolism and nutrition are central hubs to be considered when patients are enrolled in these therapies because these factors might modulate or play a role in the patients’ outcome, as they are well-recognized immune-modulating effector factors [317,318,319].

Recently, metabolism and diet have been identified as key modulators of CAR-T efficacy, reflecting the intricacy of the underlying molecular mechanisms involved in CAR-T therapies and adding an initially unexpected layer of complexity [320,321].

Despite enormous progress in T-cell generation and manufacturing, technical challenges remain in ex vivo manufacturing, T-cell purity and activation, logistics limitations, and cost/reimbursement considerations that need refinement in this stimulating and expanding field of immunotherapy [191,192].

Overall, CAR-T therapies are transitioning from specialized anticancer agents to modular platforms for immune system enhancement, and their future success will depend on balancing effectiveness, controllability, and safety.

## Figures and Tables

**Table 1:** CAR-T costimulatory modules of second and third generations.

Co-Stimulatory Domain	Alternative Name	Typical Use in CAR Design	Representative Reference
CD28	—	Second-generation CARs; clinically established	Kowolik et al., *Cancer Research* (2006) [152]
4-1BB	CD137; TNFRSF9	Second-generation CARs; clinically established	Song et al., *Cancer Research* (2011) [153]
ICOS	CD278	Second-/third-generation; preclinical/translational	Guedan et al., *Blood* (2014) [154]
OX40	CD134; TNFRSF4	Second-/third-generation; preclinical/translational	Hombach et al., *OncoImmunology* (2012) [155]
CD27	TNFRSF7	Second-/third-generation; preclinical/translational	Song et al., *Blood* (2012) [156]

**Table 2:** CAR-NK costimulatory modules of first and second generations.

Co-Stimulatory Domain	Alternative Name	Typical Use in CAR Design	Representative Reference
CD28	—	Borrowed from CAR-T designs; used in some first-/second-generation CAR-NK constructs	Kok et al., *iScience* (2025) [157]
4-1BB	CD137; TNFRSF9	Borrowed from CAR-T designs; used in conventional CAR-NK constructs	Bashiri Dezfouli et al., *Cells* (2021) [158]
2B4	CD244; SLAMF4	NK-tailored co-stimulatory domain; widely used in optimized CAR-NK designs	Li et al., *Cell Stem Cell* (2018) [159]
DAP10	HCST	NK adaptor/signaling module; often combined with 2B4 in NK-optimized CARs	Yi et al., *J Exp Clin Cancer Res* (2025) [160]
DAP12	TYROBP; KARAP	NK adaptor/signaling module; strong activating backbone in CAR-NK designs	Töpfer et al., *J Immunol* (2015) [161]
DNAM1	CD226	NK-associated co-stimulatory domain; tested alone or with 2B4	Huang et al., *Cancer Manag Res* (2020) [162]

**Table 3:** Comparative description of CAR strategies. Description of the main CAR strategies indicating the main features of each type.

Feature	CAR-T Cells	CAR-NK Cells	CAR-Macrophages/CAR-Mφ
**Cell type**	Modified T lymphocytes.	Modified NK cells.	Modified macrophages.
**General principle**	Redirect T-cell cytotoxicity against tumor antigens.	Combine CAR targeting with innate NK cytotoxicity.	Redirect phagocytic and immunomodulatory functions toward the tumor.
**Antitumor mechanism**	Direct elimination of tumor cells by activated T-cells.	CAR-dependent cytotoxicity and natural NK cytotoxicity.	Phagocytosis, antigen presentation, and activation of T/NK responses.
**Architecture and targets**	CARs with scFv, transmembrane domain, and intracellular signaling; CD19, CD22, NKG2D, and advanced generations.	NK-adapted CARs; IL-15, CXCR4, and NKG2D-CAR-NK.	CAR-Mφ with macrophage activation modules; CD19, CD22, HER2, mesothelin, anti-ALK, and CD47/HER2.
**Advantages**	High efficacy in B-cell hematological tumors; highly developed platform.	“Off-the-shelf” potential, lower GvHD, and reduced toxicity.	Particularly relevant for solid tumors and TME remodeling.
**Limitations**	Toxicity, antigen escape, exhaustion, and low efficacy in solid tumors.	Less developed, limited persistence, and insufficient tumor infiltration.	Early-stage development, complex manufacturing, and need to control immune activation.
**Clinical status**	Consolidated in CD19^+^ hematological malignancies; new generations under development.	Emerging, with trials in B-cell malignancies and ongoing optimization.	Emerging/preclinical–early clinical, mainly focused on solid tumors.

## Data Availability

No new data were created or analyzed in this study. Data sharing is not applicable to this article.

## References

[1] Kellie S., Al-Mansour Z. (2017). Overview of the Immune System. Micro-Nanotechnol. Vaccine Dev..

[2] Paul S., Lal G. (2017). The molecular mechanism of natural killer cells function and its importance in cancer immunotherapy. Front. Immunol..

[3] Bernarreggi D., Pouyanfard S., Kaufman D.S. (2019). Development of innate immune cells from human pluripotent stem cells. Exp. Hematol..

[4] Demaria O., Cornen S., Daëron M., Morel Y., Medzhitov R., Vivier E. (2019). Harnessing innate immunity in cancer therapy. Nature.

[5] Osmond D.G. (1985). The Ontogeny and Organization of the Lymphoid System. J. Investig. Dermatol..

[6] Chinn I.K., Blackburn C.C., Manley N.R., Sempowski G.D. (2012). Changes in Primary Lymphoid Organs with Aging. Semin. Immunol..

[7] Blom B., Spits H. (2006). Development of human lymphoid cells. Annu. Rev. Immunol..

[8] Ruddle N.H., Akirav E.M. (2009). Secondary Lymphoid Organs: Responding to Genetic and Environmental Cues in Ontogeny and the Immune Response. J. Immunol..

[9] Courtney A.H., Lo W.-L., Weiss A. (2018). TCR Signaling: Mechanisms of Initiation and Propagation. Trends Biochem. Sci..

[10] Palmer E. (2003). Negative selection—Clearing out the bad apples from the T-cell repertoire. Nat. Rev. Immunol..

[11] Zygmunt B., Veldhoen M. (2011). T Helper Cell Differentiation: More than Just Cytokines. Adv. Immunol..

[12] Ivanova E.A., Orekhov A.N. (2015). T Helper Lymphocyte Subsets and Plasticity in Autoimmunity and Cancer: An Overview. BioMed Res. Int..

[13] Hirahara K., Poholek A., Vahedi G., Laurence A., Kanno Y., Milner J.D., O’sHea J.J. (2013). Mechanisms underlying helper T-cell plasticity: Implications for immune-mediated disease. J. Allergy Clin. Immunol..

[14] Kidd P. (2003). Th1/Th2 balance: The hypothesis, its limitations, and implications for health and disease. Altern. Med. Rev..

[15] Kanagaratham C., El Ansari Y.S., Lewis O.L., Oettgen H.C. (2020). IgE and IgG Antibodies as Regulators of Mast Cell and Basophil Functions in Food Allergy. Front. Immunol..

[16] Farhood B., Najafi M., Mortezaee K. (2019). CD8+ cytotoxic T lymphocytes in cancer immunotherapy: A review. J. Cell. Physiol..

[17] Bossi G., Griffiths G.M. (2005). CTL secretory lysosomes: Biogenesis and secretion of a harmful organelle. Semin. Immunol..

[18] Voskoboinik I., Whisstock J.C., Trapani J.A. (2015). Perforin and granzymes: Function, dysfunction and human pathology. Nat. Rev. Immunol..

[19] Martinon F., Chen X., Lee A.H., Glimcher L.H. (2010). TLR activation of the transcription factor XBP1 regulates innate immune responses in macrophages. Nat. Immunol..

[20] Golstein P., Griffiths G.M. (2018). An early history of T cell-mediated cytotoxicity. Nat. Rev. Immunol..

[21] Andersen M.H., Schrama D., Thor Straten P., Becker J.C. (2006). Cytotoxic T cells. J. Investig. Dermatol..

[22] Magalhaes J.G., Philpott D.J., Nahori M., Jéhanno M., Fritz J., Bourhis L., Viala J., Hugot J., Giovannini M., Bertin J. (2005). Murine Nod1 but not its human orthologue mediates innate immune detection of tracheal cytotoxin. EMBO Rep..

[23] Ollila J., Vihinen M. (2005). B-cells. Int. J. Biochem. Cell Biol..

[24] Clark R., Kupper T. (2005). Old Meets New: The Interaction Between Innate and Adaptive Immunity. J. Investig. Dermatol..

[25] Moreno-Angarita A., Aragón C.C., Tobón G.J. (2020). Cathelicidin LL-37: A new important molecule in the pathophysiology of systemic lupus erythematosus. J. Transl. Autoimmun..

[26] De Yang B., Chen Q., Schmidt A.P., Anderson G.M., Wang J.M., Wooters J., Oppenheim J.J., Chertov O. (2000). LL-37, the neutrophil granule- and epithelial cell-derived cathelicidin, utilizes formyl peptide receptor-like 1 (FPRL1) as a receptor to chemoattract human peripheral blood neutrophils, monocytes, and T cells. J. Exp. Med..

[27] Borowczyk J., Shutova M., Brembilla N.C., Boehncke W.H. (2021). IL-25 (IL-17E) in epithelial immunology and pathophysiology. J. Allergy Clin. Immunol..

[28] Stanbery A.G., Smita S., von Moltke J., Tait Wojno E.D., Ziegler S.F. (2022). TSLP, IL-33, and IL-25: Not just for allergy and helminth infection. J. Allergy Clin. Immunol..

[29] Hong H., Liao S., Chen F., Yang Q., Wang D.Y. (2020). Role of IL-25, IL-33, and TSLP in triggering united airway diseases toward type 2 inflammation. Allergy.

[30] Kaufmann S.H.E. (2008). Paul Ehrlich: Founder of chemotherapy. Nat. Rev. Drug Discov..

[31] Ehrlich P., Himmelweit F. (1960). Experimental Researches on Specific Therapy: On Immunity with special Reference to the Relationship between Distribution and Action of Antigens the Harben Lectures for 1907 of the Royal Institute of Public Hea. The Collected Papers of Paul Ehrlich.

[32] Gach-Janczak K., Drogosz-Stachowicz J., Janecka A., Wtorek K., Mirowski M. (2024). Historical Perspective and Current Trends in Anticancer Drug Development. Cancers.

[33] Monneret C. (2011). Platinum anticancer drugs. From serendipity to rational design. Ann. Pharm. Fr..

[34] Chabner B.A., Roberts T.G. (2005). Chemotherapy and the war on cancer. Nat. Rev. Cancer.

[35] Povo-Retana A., Landauro-Vera R., Alvarez-Lucena C., Cascante M., Boscá L. (2024). Trabectedin and Lurbinectedin Modulate the Interplay between Cells in the Tumour Microenvironment—Progresses in Their Use in Combined Cancer Therapy. Molecules.

[36] Bonadonna G., Santoro A. (1982). Evolution in the Treatment Strategy of Hodgkin’S Disease. Adv. Cancer Res..

[37] Longo D.L., Young R.C., Wesley M., Hubbard S.M., Duffey P.L., Jaffe E.S., DeVita V.T. (1986). Twenty years of MOPP therapy for Hodgkin’s disease. J. Clin. Oncol..

[38] Zhang X., Zhu L., Zhang H., Chen S., Xiao Y. (2022). CAR-T Cell Therapy in Hematological Malignancies: Current Opportunities and Challenges. Front. Immunol..

[39] Amini L., Silbert S.K., Maude S.L., Nastoupil L.J., Ramos C.A., Brentjens R.J., Sauter C.S., Shah N.N. (2022). Preparing for CAR T cell therapy: Patient selection, bridging therapies and lymphodepletion. Nat. Rev. Clin. Oncol..

[40] Chan A.C., Martyn G.D., Carter P.J. (2025). Fifty years of monoclonals: The past, present and future of antibody therapeutics. Nat. Rev. Immunol..

[41] Diehl V., Thomas R.K., Re D. (2004). Review Part II: Hodgkin’s lymphoma—Diagnosis and treatment. Lancet Oncol..

[42] Ansell S.M. (2024). Hodgkin lymphoma: 2025 update on diagnosis, risk-stratification, and management. Am. J. Hematol..

[43] Che Y., Ding X., Xu L., Zhao J., Zhang X., Li N., Sun X. (2023). Advances in the treatment of Hodgkin’s lymphoma (Review). Int. J. Oncol..

[44] Cook A.M., Lesterhuis W.J., Nowak A.K., Lake R.A. (2016). Chemotherapy and immunotherapy: Mapping the road ahead. Curr. Opin. Immunol..

[45] Marra A., Viale G., Curigliano G. (2019). Recent advances in triple negative breast cancer: The immunotherapy era. BMC Med..

[46] Wang C., Qiao W., Jiang Y., Zhu M., Shao J., Wang T., Liu D., Li W. (2020). The landscape of immune checkpoint inhibitor plus chemotherapy versus immunotherapy for advanced non-small-cell lung cancer: A systematic review and meta-analysis. J. Cell. Physiol..

[47] Khatami F., Aghamir S.M.K., Tavangar S.M. (2019). Oncometabolites: A new insight for oncology. Mol. Genet. Genom. Med..

[48] Wang T.-X., Liang J.-Y., Zhang C., Xiong Y., Guan K.-L., Yuan H.-X. (2019). The oncometabolite 2-hydroxyglutarate produced by mutant IDH1 sensitizes cells to ferroptosis. Cell Death Dis..

[49] Lin M.D., Tsai A.C.Y., Abdullah K.G., McBrayer S.K., Shi D.D. (2024). Treatment of IDH-mutant glioma in the INDIGO era. npj Precis. Oncol..

[50] Mellinghoff I.K., Bent M.J.v.D., Blumenthal D.T., Touat M., Peters K.B., Clarke J., Mendez J., Yust-Katz S., Welsh L., Mason W.P. (2023). Vorasidenib in IDH1- or IDH2-Mutant Low-Grade Glioma. N. Engl. J. Med..

[51] Abbas A.K., Litchman A.H., Pillai S. (2018). Cellular and Molecular Immunology.

[52] Pavón Romero L., Jiménez Martínez M.C., Garcés Álvarez M.E. (2016). Inmunología Molecular, Celular Y Translacional.

[53] Plotkin S.A. (2005). Vaccines: Past, present and future. Nat. Med..

[54] Guérin N. (2007). Histoire de la vaccination: De l’empirisme aux vaccins recombinants. Rev. Med. Interne.

[55] Plotkin S.A. (2009). Vaccines: The fourth century. Clin. Vaccine Immunol..

[56] Obeid E., Nanda R., Fu Y.X., Olopade O.I. (2013). The role of tumor-associated macrophages in breast cancer progression (review). Int. J. Oncol..

[57] Abbott M., Aocnp Ò., Ustoyev Y. (2019). Seminars in Oncology Nursing Cancer and the Immune System: The History and Background of Immunotherapy. Semin. Oncol. Nurs..

[58] Oiseth S.J., Aziz M.S. (2017). Cancer immunotherapy: A brief review of the history, possibilities, and challenges ahead. J. Cancer Metastasis Treat..

[59] Tieu M.V., Pham D.T., Cho S. (2024). Bacteria-based cancer therapy: Looking forward. Biochim. Biophys. Acta Rev. Cancer.

[60] Köhler G., Milstein C. (1976). Derivation of specific antibody-producing tissue culture and tumor lines by cell fusion. Eur. J. Immunol..

[61] Köhler G., Howe S.C., Milstein C. (1976). Fusion between immunoglobulin-secreting and nonsecreting myeloma cell lines. Eur. J. Immunol..

[62] Köhler G., Milstein C. (1975). Continuous cultures of fused cells secreting antibody of predefined specificity. Nature.

[63] Eshhar Z., Waks T., Gkoss G., Schindler D.G. (1993). Specific activation and targeting of cytotoxic lymphocytes through chimeric single chains consisting of antibody-binding domains and the γ or ζ subunits of the immunoglobulin and T-cell receptors. Proc. Natl. Acad. Sci. USA.

[64] Brocker T. (2000). Chimeric Fv-ζ or Fv-ε receptors are not sufficient to induce activation or cytokine production in peripheral T cells. Blood.

[65] Acuto O., Michel F. (2003). CD28-mediated co-stimulation: A quantitative support for TCR signalling. Nat. Rev. Immunol..

[66] Finney H.M., Akbar A.N., Lawson A.D.G. (2004). Activation of Resting Human Primary T Cells with Chimeric Receptors: Costimulation from CD28, Inducible Costimulator, CD134, and CD137 in Series with Signals from the TCRζ Chain. J. Immunol..

[67] Pegram H.J., Lee J.C., Hayman E.G., Imperato G.H., Tedder T.F., Sadelain M., Brentjens R.J. (2012). Tumor-targeted T cells modified to secrete IL-12 eradicate systemic tumors without need for prior conditioning. Blood.

[68] Vormittag P., Gunn R., Ghorashian S., Veraitch F.S. (2018). A guide to manufacturing CAR T cell therapies. Curr. Opin. Biotechnol..

[69] Ma S., Li X., Wang X., Cheng L., Li Z., Zhang C., Ye Z., Qian Q. (2019). Current progress in car-t cell therapy for solid tumors. Int. J. Biol. Sci..

[70] Kagoya Y., Tanaka S., Guo T., Anczurowski M., Wang C.-H., Saso K., O Butler M., Minden M.D., Hirano N. (2018). A novel chimeric antigen receptor containing a JAK-STAT signaling domain mediates superior antitumor effects. Nat. Med..

[71] Ibáñez-Navarro M., Fernández A., Escudero A., Esteso G., Campos-Silva C., Navarro-Aguadero M.Á., Leivas A., Caracuel B.R., Rodríguez-Antolín C., Ortiz A. (2023). NKG2D-CAR memory T cells target pediatric T-cell acute lymphoblastic leukemia in vitro and in vivo but fail to eliminate leukemia initiating cells. Front. Immunol..

[72] Asmamaw Dejenie T., Medhin M.T., Terefe G.D., Admasu F.T., Tesega W.W., Abebe E.C. (2022). Current updates on generations, approvals, and clinical trials of CAR T-cell therapy. Hum. Vaccines Immunother..

[73] Mulvey A., Trueb L., Coukos G., Arber C. (2025). Novel strategies to manage CAR-T cell toxicity. Nat. Rev. Drug Discov..

[74] Ashley E.A. (2016). Towards precision medicine. Nat. Rev. Genet..

[75] Pokorska-Bocci A., Stewart A., Sagoo G.S., Hall A., Kroese M., Burton H. (2014). ‘Personalized medicine’: What’s in a name?. Pers. Med..

[76] Hodson R. (2016). Precision medicine 8. Nature.

[77] Moscow J.A., Fojo T., Schilsky R.L. (2017). Clinical evidence of the safety and efficacy of treatments, demonstrated by prospective clinical trials, provides the framework for much of oncology practice. Nat. Rev. Clin. Oncol..

[78] Seufferlein T., Mayerle J. (2016). Pancreatic cancer in 2015: Precision medicine in pancreatic cancer-fact or fiction?. Nat. Rev. Gastroenterol. Hepatol..

[79] Liu M., Yang F., Xu Y. (2022). Global Trends of Stem Cell Precision Medicine Research (2018–2022): A Bibliometric Analysis. Front. Surg..

[80] Wilkinson M.D., Dumontier M., Aalbersberg I.J., Appleton G., Axton M., Baak A., Blomberg N., Boiten J.W., da Silva Santos L.B., Bourne P.E. (2016). Comment: The FAIR Guiding Principles for scientific data management and stewardship. Sci. Data.

[81] Lekadir K., Frangi A.F., Porras A.R., Glocker B., Cintas C., Langlotz C.P., Weicken E., Asselbergs F.W., Prior F., Collins G.S. (2025). FUTURE-AI: International consensus guideline for trustworthy and deployable artificial intelligence in healthcare. BMJ.

[82] Moons K.G.M., Damen J.A., Kaul T., Hooft L., Navarro C.A., Dhiman P., Beam A.L., Van Calster B., Celi L.A., Denaxas S. (2025). PROBAST+AI: An updated quality, risk of bias, and applicability assessment tool for prediction models using regression or artificial intelligence methods. BMJ.

[83] Vaidya A., Chen R.J., Williamson D.F.K., Song A.H., Jaume G., Yang Y., Hartvigsen T., Dyer E.C., Lu M.Y., Lipkova J. (2024). Demographic bias in misdiagnosis by computational pathology models. Nat. Med..

[84] Dakkak W., Taylor A. (2017). AACR Project GENIE: Powering Precision Medicine. Physiol. Behav..

[85] Osterman T.J., Terry M., Miller R.S. (2020). Improving Cancer Data Interoperability: The Promise of the Minimal Common Oncology Data Elements (mCODE) Initiative. JCO Clin. Cancer Inform..

[86] Rehm H.L., Page A.J., Smith L., Adams J.B., Alterovitz G., Babb L.J., Barkley M.P., Baudis M., Beauvais M.J., Beck T. (2021). GA4GH: International policies and standards for data sharing across genomic research and healthcare. Cell Genom..

[87] Finlayson S.G., Subbaswamy A., Singh K., Bowers J., Kupke A., Zittrain J., Kohane I.S., Saria S. (2021). The Clinician and Dataset Shift in Artificial Intelligence. N. Engl. J. Med..

[88] Vanguri R.S., Luo J., Aukerman A.T., Egger J.V., Fong C.J., Horvat N., Pagano A., Araujo-Filho J., Geneslaw L., Rizvi H. (2022). Multimodal integration of radiology, pathology and genomics for prediction of response to PD-(L)1 blockade in patients with non-small cell lung cancer. Nat. Cancer.

[89] Chen R.J., Lu M.Y., Williamson D.F., Chen T.Y., Lipkova J., Noor Z., Shaban M., Shady M., Williams M., Joo B. (2022). Pan-cancer integrative histology-genomic analysis via multimodal deep learning. Cancer Cell.

[90] Vasey B. (2022). Reporting guideline for the early-stage clinical evaluation of decision support systems driven by artificial intelligence: DECIDE-AI. Nat. Med..

[91] Ghassemi M., Oakden-Rayner L., Beam A.L. (2021). The false hope of current approaches to explainable artificial intelligence in health care. Lancet Digit. Health.

[92] Liu X., Rivera S.C., Moher D., Calvert M.J., Denniston A.K. (2020). Reporting guidelines for clinical trial reports for interventions involving artificial intelligence: The CONSORT-AI Extension. BMJ.

[93] Rivera S.C., Liu X., Chan A.W., Denniston A.K., Calvert M.J. (2020). Guidelines for clinical trial protocols for interventions involving artificial intelligence: The SPIRIT-AI Extension. BMJ.

[94] Han R., Shakeri Z., Topol E.J. (2024). Clinical Practice: A Scoping Review. npj Digit. Med..

[95] Pollard A.J., Bijker E.M. (2021). A guide to vaccinology: From basic principles to new developments. Nat. Rev. Immunol..

[96] Vetter V., Denizer G., Friedland L.R., Krishnan J., Shapiro M. (2018). Understanding modern-day vaccines: What you need to know. Ann. Med..

[97] Kong Z., Wang Y., Ma W. (2018). Vaccination in the immunotherapy of glioblastoma. Hum. Vaccines Immunother..

[98] McCarthy E.F. (2006). The toxins of William B. Coley and the treatment of bone and soft-tissue sarcomas. Iowa Orthop. J..

[99] Clements D. (2016). Vaccination to Prevent Cancer. N. C. Med. J..

[100] Sellars M.C., Wu C.J., Fritsch E.F. (2022). Cancer vaccines: Building a bridge over troubled waters. Cell.

[101] Zhou J., Kroll A.V., Holay M., Fang R.H., Zhang L. (2020). Biomimetic Nanotechnology toward Personalized Vaccines. Adv. Mater..

[102] Zhang J., Liu J., Wang Y., Song Y., Xu Y., Zhang H., Zhou W., Wang W., Xie Y., Jin Q. (2025). Macrophage hitchhiking for smart nanomedicine: Bridging immune navigation and targeted therapy. J. Control. Release.

[103] Harari A., Graciotti M., Bassani-Sternberg M., Kandalaft L.E. (2020). Antitumour dendritic cell vaccination in a priming and boosting approach. Nat. Rev. Drug Discov..

[104] Gardner A., de Mingo Pulido Á., Ruffell B. (2020). Dendritic Cells and Their Role in Immunotherapy. Front. Immunol..

[105] Aly H.A.A. (2012). Cancer therapy and vaccination. J. Immunol. Methods.

[106] Jiang H., Wang Q., Sun X. (2017). Lymph node targeting strategies to improve vaccination efficacy. J. Control. Release.

[107] Hale D.F., Vreeland T.J., Peoples G.E. (2016). Arming the Immune System Through Vaccination to Prevent Cancer Recurrence. Am. Soc. Clin. Oncol. Educ. Book.

[108] Wang X., Niu Y., Bian F. (2025). The progress of tumor vaccines clinical trials in non-small cell lung cancer. Clin. Transl. Oncol..

[109] Magoola M., Niazi S.K. (2025). Current Progress and Future Perspectives of RNA-Based Cancer Vaccines: A 2025 Update. Cancers.

[110] Martín E., Reina G., Carlos S. (2025). Advances in Therapeutic Vaccines Against HPV: A Review of Human Clinical Trials. Curr. Oncol..

[111] Huang F.Y., Lin Y.Y., Tan G.H. (2026). Bacterialized tumor cells as vaccine. EMBO Mol. Med..

[112] Patel A., Kaufman H.L., Disis M.L. (2017). Next generation approaches for tumor vaccination. Chin. Clin. Oncol..

[113] Cortés-Selva D., Dasgupta B., Singh S., Grewal I.S. (2021). Innate and Innate-Like Cells: The Future of Chimeric Antigen Receptor (CAR) Cell Therapy. Trends Pharmacol. Sci..

[114] Martinez M., Moon E.K. (2019). CAR T Cells for Solid Tumors: New Strategies for Finding, Infiltrating, and Surviving in the Tumor Microenvironment. Front. Immunol..

[115] Filley A.C., Henriquez M., Dey M. (2018). CART immunotherapy: Development, success, and translation to malignant gliomas and other solid tumors. Front. Oncol..

[116] Sterner R.C., Sterner R.M. (2021). CAR-T cell therapy: Current limitations and potential strategies. Blood Cancer J..

[117] Huang R., Li X., He Y., Zhu W., Gao L., Liu Y., Gao L., Wen Q., Zhong J.F., Zhang C. (2020). Recent advances in CAR-T cell engineering. J. Hematol. Oncol..

[118] Okuyama N., Tamada K., Tamura H. (2016). Recent advances and future challenges in cancer immunotherapy. Rinsho Ketsueki.

[119] Wang W., Jiang J., Wu C. (2020). CAR-NK for tumor immunotherapy: Clinical transformation and future prospects. Cancer Lett..

[120] Biederstädt A., Rezvani K. (2021). Engineering the next generation of CAR-NK immunotherapies. Int. J. Hematol..

[121] Sadri M., Heidari S., Faridzadeh A., Roozbehani M., Toosi S., Mahmoudian R.A., Hoseinzadeh A., Fard M.T.S., Arab F.L., Fard S.R. (2024). Potential applications of macrophages in cancer immunotherapy. Biomed. Pharmacother..

[122] Chen Y., Yu Z., Tan X., Jiang H., Xu Z., Fang Y., Han D., Hong W., Wei W., Tu J. (2021). CAR-macrophage: A new immunotherapy candidate against solid tumors. Biomed. Pharmacother..

[123] Xia Y., Rao L., Yao H., Wang Z., Ning P., Chen X. (2020). Engineering Macrophages for Cancer Immunotherapy and Drug Delivery. Adv. Mater..

[124] Li J., Chen P., Ma W. (2024). The next frontier in immunotherapy: Potential and challenges of CAR-macrophages. Exp. Hematol. Oncol..

[125] Chettri D., Satapathy B.P., Yadav R., Uttam V., Jain A., Prakash H. (2024). CAR-macrophages: Tailoring cancer immunotherapy. Front. Immunol..

[126] Santoni M., Massari F., Montironi R., Battelli N. (2021). Manipulating macrophage polarization in cancer patients: From nanoparticles to human chimeric antigen receptor macrophages. Biochim. Biophys. Acta Rev. Cancer.

[127] Morva A., Arroyo A.B., Andreeva L., Tapia-Abellán A., Luengo-Gil G. (2025). Unleashing the power of CAR-M therapy in solid tumors: A comprehensive review. Front. Immunol..

[128] Wang Y., Lin L., Wang X., Li J., Pan Q., Kou H., Yin J., Gao F., Liao X., Zhang C. (2025). Synergically enhanced anti-tumor immunity of in vivo panCAR by circRNA vaccine boosting. Cell Rep. Med..

[129] Xin Q., Chen Y., Sun X., Li R., Wu Y., Huang X. (2024). CAR-T therapy for ovarian cancer: Recent advances and future directions. Biochem. Pharmacol..

[130] Paasch D., Lachmann N. (2024). CAR macrophages tuning the immune symphony of anti-cancer therapies. Cell Stem Cell.

[131] Shi Y., Li X., Dong Y., Yuan H., Wang Y., Yang R. (2025). Exploring the potential of CAR-macrophage therapy. Med. Rev..

[132] Wei F., Liu H., Wang Y., Li Y., Han S. (2024). Engineering macrophages and their derivatives: A new hope for antitumor therapy. Biomed. Pharmacother..

[133] Shu J., Xie W., Chen Z., Offringa R., Hu Y., Mei H. (2024). The enchanting canvas of CAR technology: Unveiling its wonders in non-neoplastic diseases. Med.

[134] Chen Y., Xin Q., Zhu M., Qiu J., Luo Y., Li R., Wei W., Tu J. (2025). Exploring CAR-macrophages in non-tumor diseases: Therapeutic potential beyond cancer. J. Adv. Res..

[135] Wang Z., Wu Z., Liu Y., Han W. (2017). New development in CAR-T cell therapy. J. Hematol. Oncol..

[136] Zhang C., Liu J., Zhong J.F., Zhang X. (2017). Engineering CAR-T cells. Biomark. Res..

[137] Tang L., Pan S., Wei X., Xu X., Wei Q. (2023). Arming CAR-T cells with cytokines and more: Innovations in the fourth-generation CAR-T development. Mol. Ther..

[138] Mishra A., Maiti R., Mohan P., Gupta P. (2024). Antigen loss following CAR-T cell therapy: Mechanisms, implications, and potential solutions. Eur. J. Haematol..

[139] Shah N.N., Fry T.J. (2019). Mechanisms of resistance to CAR T cell therapy. Nat. Rev. Clin. Oncol..

[140] Neelapu S.S., Locke F.L., Bartlett N.L., Lekakis L.J., Miklos D.B., Jacobson C.A., Braunschweig I., Oluwole O.O., Siddiqi T., Lin Y. (2017). Axicabtagene Ciloleucel CAR T-Cell Therapy in Refractory Large B-Cell Lymphoma. N. Engl. J. Med..

[141] Porter D.L., Hwang W.-T., Frey N.V., Lacey S.F., Shaw P.A., Loren A.W., Bagg A., Marcucci K.T., Shen A., Gonzalez V. (2015). Chimeric antigen receptor T cells persist and induce sustained remissions in relapsed refractory chronic lymphocytic leukemia. Sci. Transl. Med..

[142] Brudno J.N., Kochenderfer J.N. (2024). Advances in the mechanisms and management of CAR T-cell toxicities. Nat. Rev. Clin. Oncol..

[143] Labanieh L., Mackall C.L. (2023). CAR immune cells: Design principles, resistance and the next generation. Nature.

[144] Dotti G., Savoldo B., Brenner M. (2009). Fifteen years of gene therapy based on chimeric antigen receptors: Are we nearly there yet?. Hum. Gene Ther..

[145] Schuster S.J., Bishop M.R., Tam C.S., Waller E.K., Borchmann P., McGuirk J.P., Jäger U., Jaglowski S., Andreadis C., Westin J.R. (2019). Tisagenlecleucel in Adult Relapsed or Refractory Diffuse Large B-Cell Lymphoma. N. Engl. J. Med..

[146] Marin V., Pizzitola I., Agostoni V., Attianese G.M.P.G., Finney H., Lawson A., Pule M., Rousseau R., Biondi A., Biagi E. (2010). Cytokine-induced killer cells for cell therapy of acute myeloid leukemia: Improvement of their immune activity by expression of CD33-specific chimeric receptors. Haematologica.

[147] Gomes da Silva D., Mukherjee M., Madhuwanti S., Dakhova O., Liu H., Grilley B., Gee A., Neelapu S., Rooney C., Heslop H. (2016). Direct Comparison of In Vivo Fate of Second and Third-Generation CD19-Specific Chimeric Antigen Receptor (CAR)-T Cells in Patients with B-Cell Lymphoma: Reversal of Toxicity from Tonic Signaling. Blood.

[148] Chmielewski M., Abken H. (2015). TRUCKs: The fourth generation of CARs. Expert Opin. Biol. Ther..

[149] Koneru M., Purdon T.J., Spriggs D., Koneru S., Brentjens R.J. (2015). IL-12 secreting tumor-targeted chimeric antigen receptor T cells eradicate ovarian tumors in vivo. OncoImmunology.

[150] Chmielewski M., Abken H. (2020). TRUCKS, the fourth-generation CAR T cells: Current developments and clinical translation. Adv. Cell Gene Ther..

[151] Weinkove R., George P., Dasyam N., McLellan A.D. (2019). Selecting costimulatory domains for chimeric antigen receptors: Functional and clinical considerations. Clin. Transl. Immunol..

[152] Kowolik C.M., Topp M.S., Gonzalez S., Pfeiffer T., Olivares S., Gonzalez N., Smith D.D., Forman S.J., Jensen M.C., Cooper L.J. (2006). CD28 Costimulation Provided through a CD19-Specific Chimeric Antigen Receptor Enhances In vivo Persistence and Antitumor Efficacy of Adoptively Transferred T Cells. Cancer Res..

[153] Song D.-G.G., Ye Q., Carpenito C., Poussin M., Wang L.P., Ji C., Figini M., June C.H., Coukos G., Powell D.J. (2011). In Vivo Persistence, Tumor Localization, and Antitumor Activity of CAR-Engineered T Cells Is Enhanced by Costimulatory Signaling through CD137 (4-1BB). Cancer Res..

[154] Guedan S., Chen X., Madar A., Carpenito C., McGettigan S.E., Frigault M.J., Lee J., Posey A.D., Scholler J., Scholler N. (2014). ICOS-based chimeric antigen receptors program bipolar TH17/TH1 cells. Blood.

[155] Hombach A.A., Heiders J., Foppe M., Chmielewski M., Abken H. (2012). OX40 costimulation by a chimeric antigen receptor abrogates CD28 and IL-2 induced IL-10 secretion by redirected CD4+ T cells. Oncoimmunology.

[156] Song D.-G.G., Ye Q., Poussin M., Harms G.M., Figini M., Powell D.J. (2012). CD27 costimulation augments the survival and antitumor activity of redirected human T cells in vivo. Blood.

[157] Kok N., Ozkazanc D., van Vliet A.A., Steenmans D., Singh S.P., Sutlu T., Georgoudaki A.M., Raimo M., Spanholtz J., Duru A.D. (2025). CD28 signaling domain boosts persistence and in vivo anti-tumor activity of stem cell-derived CD19-CAR-NK cells. iScience.

[158] Bashiri Dezfouli A., Yazdi M., Pockley A.G., Khosravi M., Kobold S., Wagner E., Multhoff G. (2021). NK Cells Armed with Chimeric Antigen Receptors (CAR): Roadblocks to Successful Development. Cells.

[159] Li Y., Hermanson D.L., Moriarity B.S., Kaufman D.S. (2018). Human iPSC-Derived Natural Killer Cells Engineered with Chimeric Antigen Receptors Enhance Anti-tumor Activity. Cell Stem Cell.

[160] Yi E., Lee E., Park H.J., Lee H.H., Yun S.H., Kim H.S. (2025). A chimeric antigen receptor tailored to integrate complementary activation signals potentiates the antitumor activity of NK cells. J. Exp. Clin. Cancer Res..

[161] Töpfer K., Cartellieri M., Michen S., Wiedemuth R., Müller N., Lindemann D., Bachmann M., Füssel M., Schackert G., Temme A. (2015). DAP12-Based Activating Chimeric Antigen Receptor for NK Cell Tumor Immunotherapy. J. Immunol..

[162] Huang Y., Zeng J., Liu T., Xu Q., Song X., Zeng J. (2020). DNAM1 and 2B4 Costimulatory Domains Enhance the Cytotoxicity of Anti-GPC3 Chimeric Antigen Receptor-Modified Natural Killer Cells Against Hepatocellular Cancer Cells in vitro. Cancer Manag. Res..

[163] Canedo G.D.O., Roddie C., Amrolia P.J. (2025). Dual-targeting CAR T cells for B-cell acute lymphoblastic leukemia and B-cell non-Hodgkin lymphoma. Blood Adv..

[164] Ruiz-Navarro J., Fernández-Hermira S., Sanz-Fernández I., Barbeito P., Navarro-Zapata A., Pérez-Martínez A., Garcia-Gonzalo F.R., Calvo V., Izquierdo Pastor M. (2024). Formin-like 1β phosphorylation at S1086 is necessary for secretory polarized traffic of exosomes at the immune synapse in Jurkat T lymphocytes. eLife.

[165] Cui W., Zhang X., Li Z., Dai H., Yin J., Cui Q., Liu S., Kang L., Yu L., Wu D. (2023). Long-term follow-up of tandem CD19/CD22 CAR T-Cells in r/r B-ALL patients with high-risk features. Am. J. Hematol..

[166] González-Martínez B., Galán-Gómez V., Navarro-Zapata A., Mirones-Aguilar I., Cobo M., Pernas-Sánchez A., Vallejo S., Sánchez-Zapardiel E., León-Triana O., Echecopar C. (2025). Tandem CD19/CD22 CAR T-cells as potential therapy for children and young adults with high-risk r/r B-ALL. EBioMedicine.

[167] Rafiq S., Hackett C.S., Brentjens R.J. (2020). Engineering strategies to overcome the current roadblocks in CAR T cell therapy. Nat. Rev. Clin. Oncol..

[168] Shahid Z., Jain T., Dioverti V., Pennisi M., Mikkilineni L., Thiruvengadam S.K., Shah N.N., Dadwal S., Papanicolaou G., Hamadani M. (2024). Best Practice Considerations by The American Society of Transplant and Cellular Therapy: Infection Prevention and Management After Chimeric Antigen Receptor T Cell Therapy for Hematological Malignancies. Transplant. Cell. Ther..

[169] Wensveen F.M., Jelenčić V., Polić B. (2018). NKG2D: A master regulator of immune cell responsiveness. Front. Immunol..

[170] Lanier L.L. (2015). NKG2D receptor and its ligands in host defense. Cancer Immunol. Res..

[171] Fang F., Xiao W., Tian Z. (2017). NK cell-based immunotherapy for cancer. Semin. Immunol..

[172] Xie G., Dong H., Liang Y., Ham J.D., Rizwan R., Chen J. (2020). CAR-NK cells: A promising cellular immunotherapy for cancer. EBioMedicine.

[173] Yang M.Q., Zhang S.-L., Sun L., Huang L.-T., Yu J., Zhang J.-H., Tian Y., Han C.-B., Ma J.-T. (2024). Targeting mitochondria: Restoring the antitumor efficacy of exhausted T cells. Mol. Cancer.

[174] Wang D.R., Dou L.Y., Sui L.H., Xue Y., Xu S. (2024). Natural killer cells in cancer immunotherapy. MedComm.

[175] Zhou Y., Wei S., Xu M., Wu X., Dou W., Li H., Zhang Z., Zhang S. (2024). CAR-T cell therapy for hepatocellular carcinoma: Current trends and challenges. Front. Immunol..

[176] Saito S., Nakazawa Y. (2024). CAR-T cell therapy in AML: Recent progress and future perspectives. Int. J. Hematol..

[177] Zhang Y., Zhang C., He M., Xing W., Hou R., Zhang H. (2024). Co-expression of IL-21-Enhanced NKG2D CAR-NK cell therapy for lung cancer. BMC Cancer.

[178] Guo C., Dong M., Wang X., Yu J., Jin X., Cheng S., Cui F., Qian Y., Bao Q., Zhi L. (2024). A novel MICA/B-targeted chimeric antigen receptor augments the cytotoxicity of NK cells against tumor cells. Biochem. Biophys. Res. Commun..

[179] Dagher O.K., Posey A.D. (2023). Forks in the road for CAR T and CAR NK cell cancer therapies. Nat. Immunol..

[180] Rosa R., Liu J., Lu C., Abou-El-Enein M., Murad J.P., Priceman S.J. (2026). Current state of CAR-T cell therapies for solid tumors. Med.

[181] Khan S.H., Choi Y., Veena M., Lee J.K., Shin D.S. (2024). Advances in CAR T cell therapy: Antigen selection, modifications, and current trials for solid tumors. Front. Immunol..

[182] Van Bruggen J.A.C., Martens A.J., Fraietta J., Hofland T., Tonino S., Eldering E., Levin M., Siska P., Endstra S., Rathmell J. (2019). Chronic lymphocytic leukemia cells impair mitochondrial fitness in CD8+ T cells and impede CAR T-cell efficacy. Blood.

[183] Kates M., Saibil S.D. (2024). Reprogramming T-cell metabolism to enhance adoptive cell therapies. Int. Immunol..

[184] Dumauthioz N., Tschumi B., Wenes M., Marti B., Wang H., Franco F., Li W., Lopez-Mejia I.C., Fajas L., Ho P.-C. (2021). Enforced PGC-1α expression promotes CD8 T cell fitness, memory formation and antitumor immunity. Cell. Mol. Immunol..

[185] Scharping N.E., Menk A.V., Moreci R.S., Whetstone R.D., Dadey R.E., Watkins S.C., Ferris R.L., Delgoffe G.M. (2016). The Tumor Microenvironment Represses T Cell Mitochondrial Biogenesis to Drive Intratumoral T Cell Metabolic Insufficiency and Dysfunction. Immunity.

[186] Menk A.V., Scharping N.E., Rivadeneira D.B., Calderon M.J., Watson M.J., Dunstane D., Watkins S.C., Delgoffe G.M. (2018). 4-1BB costimulation induces T cell mitochondrial function and biogenesis enabling cancer immunotherapeutic responses. J. Exp. Med..

[187] Fultang L., Booth S., Yogev O., da Costa B.M., Tubb V., Panetti S., Stavrou V., Scarpa U., Jankevics A., Lloyd G. (2020). Metabolic engineering against the arginine microenvironment enhances CAR-T cell proliferation and therapeutic activity. Blood.

[188] Ibiza S., Víctor V.M., Boscá I., Ortega A., Urzainqui A., O’COnnor J.E., Sánchez-Madrid F., Esplugues J.V., Serrador J.M. (2006). Endothelial Nitric Oxide Synthase Regulates T Cell Receptor Signaling at the Immunological Synapse. Immunity.

[189] Amitrano A.M., Kim M. (2023). Metabolic Challenges in Anticancer CD8 T Cell Functions. Immune Netw..

[190] Yun K., Siegler E.L., Kenderian S.S. (2023). Who wins the combat, CAR or TCR?. Leukemia.

[191] Guaza-Lasheras M., Nimmerfroh J., Schwarz D., de Grujil T.D., Hartmann J., Klein C., Läubli H., Lotem M., Mittelstaet J., Paget C. (2026). The evolving role of cytokines for CAR-T cell manufacturing and beyond. Nat. Biomed. Eng..

[192] Ayala Ceja M., Khericha M., Harris C.M., Puig-Saus C., Chen Y.Y. (2024). CAR-T cell manufacturing: Major process parameters and next-generation strategies. J. Exp. Med..

[193] Roda-Navarro P., Álvarez-Vallina L. (2020). Understanding the Spatial Topology of Artificial Immunological Synapses Assembled in T Cell-Redirecting Strategies: A Major Issue in Cancer Immunotherapy. Front. Cell Dev. Biol..

[194] Davenport A.J., Cross R.S., Watson K.A., Liao Y., Shi W., Prince H.M., Beavis P.A., Trapani J.A., Kershaw M.H., Ritchie D.S. (2018). Chimeric antigen receptor T cells form nonclassical and potent immune synapses driving rapid cytotoxicity. Proc. Natl. Acad. Sci. USA.

[195] Xu X., Chen H., Ren Z., Xu X., Wu W., Yang H., Wang J., Zhang Y., Zhou Q., Li H. (2024). Phase separation of chimeric antigen receptor promotes immunological synapse maturation and persistent cytotoxicity. Immunity.

[196] Xiong W., Chen Y., Kang X., Chen Z., Zheng P., Hsu Y.-H., Jang J.H., Qin L., Liu H., Dotti G. (2018). Immunological Synapse Predicts Effectiveness of Chimeric Antigen Receptor Cells. Mol. Ther..

[197] Naghizadeh A., Tsao W.-C., Cho J.H., Xu H., Mohamed M., Li D., Xiong W., Metaxas D., Ramos C.A., Liu D. (2022). In vitro machine learning-based CAR T immunological synapse quality measurements correlate with patient clinical outcomes. PLoS Comput. Biol..

[198] Cassioli C., Patrussi L., Valitutti S., Baldari C.T. (2022). Learning from TCR Signaling and Immunological Synapse Assembly to Build New Chimeric Antigen Receptors (CARs). Int. J. Mol. Sci..

[199] Jiang P., Jing S., Sheng G., Jia F. (2024). The basic biology of NK cells and its application in tumor immunotherapy. Front. Immunol..

[200] Pan K., Farrukh H., Chittepu V.C.S.R., Xu H., Pan C.-X., Zhu Z. (2022). CAR race to cancer immunotherapy: From CAR T, CAR NK to CAR macrophage therapy. J. Exp. Clin. Cancer Res..

[201] Zhang L., Meng Y., Feng X., Han Z. (2022). CAR-NK cells for cancer immunotherapy: From bench to bedside. Biomark. Res..

[202] Marin D., Li Y., Basar R., Rafei H., Daher M., Dou J., Mohanty V., Dede M., Nieto Y., Uprety N. (2024). Safety, efficacy and determinants of response of allogeneic CD19-specific CAR-NK cells in CD19+ B-cell tumors: A phase 1/2 trial. Nat. Med..

[203] Rana S., Thomas L., Kirkham A.M., Shorr R., Visram A., Maganti H., Seftel M., Allan D.S. (2025). CAR-NK cells to treat patients with cancer: A systematic scoping review of published studies and registered clinical trials. Cytotherapy.

[204] Kilgour M.K., Bastin D.J., Lee S.-H., Ardolino M., McComb S., Visram A. (2023). Advancements in CAR-NK therapy: Lessons to be learned from CAR-T therapy. Front. Immunol..

[205] Ruppel K.E., Fricke S., Köhl U., Schmiedel D. (2022). Taking Lessons from CAR-T Cells and Going Beyond: Tailoring Design and Signaling for CAR-NK Cells in Cancer Therapy. Front. Immunol..

[206] Ferrari S.M., Fallahi P., Galdiero M.R., Ruffilli I., Elia G., Ragusa F., Paparo S.R., Patrizio A., Mazzi V., Varricchi G. (2019). Immune and inflammatory cells in thyroid cancer microenvironment. Int. J. Mol. Sci..

[207] Jaiswal S., Chao M.P., Majeti R., Weissman I.L. (2010). Macrophages as mediators of tumor immunosurveillance. Trends Immunol..

[208] Bonavita E., Galdiero M.R., Jaillon S., Mantovani A. (2015). Phagocytes as Corrupted Policemen in Cancer-Related Inflammation. Adv. Cancer Res..

[209] Rabold K., Netea M.G., Adema G.J., Netea-Maier R.T. (2017). Cellular metabolism of tumor-associated macrophages—Functional impact and consequences. FEBS Lett..

[210] Liu Y., Xu R., Gu H., Zhang E., Qu J., Cao W., Huang X., Yan H., He J., Cai Z. (2021). Metabolic reprogramming in macrophage responses. Biomark. Res..

[211] Zhang Y., Sime W., Juhas M., Sjolander A. (2013). Crosstalk between colon cancer cells and macrophages via inflammatory mediators and CD47 promotes tumour cell migration. Eur. J. Cancer.

[212] Saio M., Radoja S., Marino M., Frey A.B. (2001). Tumor-infiltrating macrophages induce apoptosis in activated CD8(+) T cells by a mechanism requiring cell contact and mediated by both the cell-associated form of TNF and nitric oxide. J. Immunol..

[213] Hanahan D. (2022). Hallmarks of Cancer: New Dimensions. Cancer Discov..

[214] Hanahan D., Weinberg R.A. (2011). Hallmarks of cancer: The next generation. Cell.

[215] Senga S.S., Grose R.P. (2021). Hallmarks of cancer—The new testament. Open Biol..

[216] Hanahan D., Coussens L.M. (2012). Accessories to the Crime: Functions of Cells Recruited to the Tumor Microenvironment. Cancer Cell.

[217] Pavlova N.N., Zhu J., Thompson C.B. (2022). The hallmarks of cancer metabolism: Still emerging. Cell Metab..

[218] Colombo M.P., Mantovani A. (2005). Targeting myelomonocytic cells to revert inflammation-dependent cancer promotion. Cancer Res..

[219] Lewis C.E., Pollard J.W. (2006). Distinct role of macrophages in different tumor microenvironments. Cancer Res..

[220] Zhou J., Tang Z., Gao S., Li C., Feng Y., Zhou X. (2020). Tumor-Associated Macrophages: Recent Insights and Therapies. Front. Oncol..

[221] Petty A.J., Owen D.H., Yang Y., Huang X. (2021). Targeting Tumor-Associated Macrophages in Cancer Immunotherapy. Cancers.

[222] Christofides A., Strauss L., Yeo A., Cao C., Charest A., Boussiotis V.A. (2022). The complex role of tumor-infiltrating macrophages. Nat. Rev. Clin. Oncol..

[223] Wang H., Yung M.M.H., Ngan H.Y.S., Chan K.K.L., Chan D.W. (2021). The Impact of the Tumor Microenvironment on Macrophage Polarization in Cancer Metastatic Progression. Int. J. Mol. Sci..

[224] Fu L.Q., Du W.-L., Cai M.-H., Yao J.-Y., Zhao Y.-Y., Mou X.-Z. (2020). The roles of tumor-associated macrophages in tumor angiogenesis and metastasis. Cell. Immunol..

[225] Mehta A.K., Kadel S., Townsend M.G., Oliwa M., Guerriero J.L. (2021). Macrophage Biology and Mechanisms of Immune Suppression in Breast Cancer. Front. Immunol..

[226] Mehraj U., Dar A.H., Wani N.A., Mir M.A. (2021). Tumor microenvironment promotes breast cancer chemoresistance. Cancer Chemother. Pharmacol..

[227] Rogers T.L., Holen I. (2011). Tumour macrophages as potential targets of bisphosphonates. J. Transl. Med..

[228] Larionova I., Tuguzbaeva G., Ponomaryova A., Stakheyeva M., Cherdyntseva N., Pavlov V., Choinzonov E., Kzhyshkowska J. (2020). Tumor-Associated Macrophages in Human Breast, Colorectal, Lung, Ovarian and Prostate Cancers. Front. Oncol..

[229] Boudin L., Patient M., Romeo E., Bladé J.S., de Lesquen H. (2021). Lurbinectedin for metastatic small-cell bladder carcinoma. Eur. J. Cancer.

[230] Kristeleit R., Moreno V., Boni V., Guerra E.M., Kahatt C., Romero I., Calvo E., Basté N., A López-Vilariño J., Siguero M. (2021). Doxorubicin plus lurbinectedin in patients with advanced endometrial cancer: Results from an expanded phase i study. Int. J. Gynecol. Cancer.

[231] Oya Y., Hayakawa Y., Koike K. (2020). Tumor microenvironment in gastric cancers. Cancer Sci..

[232] Gambardella V., Castillo J., Tarazona N., Gimeno-Valiente F., Martínez-Ciarpaglini C., Cabeza-Segura M., Roselló S., Roda D., Huerta M., Cervantes A. (2020). The role of tumor-associated macrophages in gastric cancer development and their potential as a therapeutic target. Cancer Treat. Rev..

[233] Wang H., Tian T., Zhang J. (2021). Tumor-Associated Macrophages (TAMs) in Colorectal Cancer (CRC): From Mechanism to Therapy and Prognosis. Int. J. Mol. Sci..

[234] Nowak M., Klink M. (2020). The Role of Tumor-Associated Macrophages in the Progression and Chemoresistance of Ovarian Cancer. Cells.

[235] Mielgo A., Schmid M.C. (2013). Impact of tumour associated macrophages in pancreatic cancer. BMB Rep..

[236] Capece D., Fischietti M., Verzella D., Gaggiano A., Cicciarelli G., Tessitore A., Zazzeroni F., Alesse E. (2013). The Inflammatory Microenvironment in Hepatocellular Carcinoma: A Pivotal Role for Tumor-Associated Macrophages. BioMed Res. Int..

[237] Schulz M., Sevenich L. (2021). TAMs in Brain Metastasis: Molecular Signatures in Mouse and Man. Front. Immunol..

[238] Fujiwara T., Healey J., Ogura K., Yoshida A., Kondo H., Hata T., Kure M., Tazawa H., Nakata E., Kunisada T. (2021). Role of tumor-associated macrophages in sarcomas. Cancers.

[239] Li Y., You M.J., Yang Y., Hu D., Tian C. (2020). The role of tumor-associated macrophages in leukemia. Acta Haematol..

[240] Vitale I., Manic G., Coussens L.M., Kroemer G., Galluzzi L. (2019). Macrophages and Metabolism in the Tumor Microenvironment. Cell Metab..

[241] Tan Y., Wang M., Zhang Y., Ge S., Zhong F., Xia G., Sun C. (2021). Tumor-Associated Macrophages: A Potential Target for Cancer Therapy. Front. Oncol..

[242] Wu K., Lin K., Li X., Yuan X., Xu P., Ni P., Xu D. (2020). Redefining Tumor-Associated Macrophage Subpopulations and Functions in the Tumor Microenvironment. Front. Immunol..

[243] Lewis C.E., Harney A.S., Pollard J.W. (2016). The Multifaceted Role of Perivascular Macrophages in Tumors. Cancer Cell.

[244] Mantovani A., Marchesi F., Jaillon S., Garlanda C., Allavena P. (2021). Tumor-associated myeloid cells: Diversity and therapeutic targeting. Cell. Mol. Immunol..

[245] Locati M., Curtale G., Mantovani A. (2020). Diversity, Mechanisms, and Significance of Macrophage Plasticity. Annu. Rev. Pathol. Mech. Dis..

[246] Wu L., Zhang X.H.F. (2020). Tumor-Associated Neutrophils and Macrophages—Heterogenous but Not Chaotic. Front. Immunol..

[247] Nakamura K., Smyth M.J. (2020). Myeloid immunosuppression and immune checkpoints in the tumor microenvironment. Cell. Mol. Immunol..

[248] Allavena P., Mantovani A. (2012). Immunology in the clinic review series; focus on cancer: Tumour-associated macrophages: Undisputed stars of the inflammatory tumour microenvironment. Clin. Exp. Immunol..

[249] Lakshmi Narendra B., Eshvendar Reddy K., Shantikumar S., Ramakrishna S. (2013). Immune system: A double-edged sword in cancer. Inflamm. Res..

[250] Monferrer E., Sanegre S., Vieco-Martí I., López-Carrasco A., Fariñas F., Villatoro A., Abanades S., Mañes S., de la Cruz-Merino L., Noguera R. (2021). Immunometabolism modulation in therapy. Biomedicines.

[251] Millrud C.R., Bergenfelz C., Leandersson K. (2017). On the origin of myeloid-derived suppressor cells. Oncotarget.

[252] Ren H., Ma L., Jiang X., Lu Z. (2026). Immune evasion by macrophage-derived lactate. Nat. Cell Biol..

[253] Zong Z., Ren J., Yang B., Zhang L., Zhou F. (2025). Emerging roles of lysine lactyltransferases and lactylation. Nat. Cell Biol..

[254] Gordon S.R., Maute R.L., Dulken B.W., Hutter G., George B.M., McCracken M.N., Gupta R., Tsai J.M., Sinha R., Corey D. (2017). PD-1 expression by tumour-associated macrophages inhibits phagocytosis and tumour immunity. Nature.

[255] Puthenveetil A., Dubey S. (2020). Metabolic reprograming of tumor-associated macrophages. Ann. Transl. Med..

[256] Pan Y., Yu Y., Wang X., Zhang T. (2020). Tumor-Associated Macrophages in Tumor Immunity. Front. Immunol..

[257] Allavena P., Digifico E., Belgiovine C. (2021). Macrophages and cancer stem cells: A malevolent alliance. Mol. Med..

[258] Anderson N.R., Minutolo N.G., Gill S., Klichinsky M. (2021). Macrophage-based approaches for cancer immunotherapy. Cancer Res..

[259] Kadomoto S., Izumi K., Mizokami A. (2022). Macrophage Polarity and Disease Control. Int. J. Mol. Sci..

[260] Chen Y., Jin H., Song Y., Huang T., Cao J., Tang Q., Zou Z. (2021). Targeting tumor-associated macrophages: A potential treatment for solid tumors. J. Cell. Physiol..

[261] Bronte V., Brandau S., Chen S.-H., Colombo M.P., Frey A.B., Greten T.F., Mandruzzato S., Murray P.J., Ochoa A., Ostrand-Rosenberg S. (2016). Recommendations for myeloid-derived suppressor cell nomenclature and characterization standards. Nat. Commun..

[262] Corzo C.A., Condamine T., Lu L., Cotter M.J., Youn J.-I., Cheng P., Cho H.-I., Celis E., Quiceno D.G., Padhya T. (2010). HIF-1α regulates function and differentiation of myeloid-derived suppressor cells in the tumor microenvironment. J. Exp. Med..

[263] Sinha P., Clements V.K., Bunt S.K., Albelda S.M., Ostrand-Rosenberg S. (2007). Cross-talk between myeloid-derived suppressor cells and macrophages subverts tumor immunity toward a type 2 response. J. Immunol..

[264] Wang C., Zhang Q., Huang J., Lin F., Zhao D., Mu Y., Tong J., Li J., Liang Y., Zeng T. (2026). A CD138+ tumor-associated macrophage/Siglec-F+ neutrophil feed-forward loop promotes immune evasion in pancreatic cancer. J. Clin. Investig..

[265] Labelle M., Begum S., Hynes R.O. (2014). Platelets guide the formation of early metastatic niches. Proc. Natl. Acad. Sci. USA.

[266] Rachidi S., Metelli A., Riesenberg B., Wu B.X., Nelson M.H., Wallace C., Paulos C.M., Rubinstein M.P., Garrett-Mayer E., Hennig M. (2017). Platelets subvert T cell immunity against cancer via GARP-TGFβ axis. Sci. Immunol..

[267] Teijeira Á., Garasa S., Gato M., Alfaro C., Migueliz I., Cirella A., de Andrea C., Ochoa M.C., Otano I., Etxeberria I. (2020). CXCR1 and CXCR2 Chemokine Receptor Agonists Produced by Tumors Induce Neutrophil Extracellular Traps that Interfere with Immune Cytotoxicity. Immunity.

[268] Belz L., Heuvel D.v.D., Kilani B., Martinez-Navarro A., Akhalkatsi A., Joppich M., Zhu Y., Ding Q., Wan J., Stermann J. (2026). Neutrophil-Driven Trogocytosis in Pancreatic Ductal Adenocarcinoma Liver Metastases Restrains Immunosuppressive Macrophage Polarization and Limits Metastatic Progression. Gastroenterology.

[269] Zuo Y., Vohwinkel D.J., Dong B., McDowell J.R., Guzman B.V., Pandian T.S.M., Chattopadhyay S., McGray A., Olejniczak S.H., Ohm J. (2025). IL-36γ armored CAR T cells reprogram neutrophils to induce endogenous antitumor immunity. Cancer Cell.

[270] Reinfeld B.I., Madden M.Z., Wolf M.M., Chytil A., Bader J.E., Patterson A.R., Sugiura A., Cohen A.S., Ali A., Do B.T. (2021). Cell-programmed nutrient partitioning in the tumour microenvironment. Nature.

[271] Wenes M., Shang M., Di Matteo M., Goveia J., Martín-Pérez R., Serneels J., Prenen H., Ghesquière B., Carmeliet P., Mazzone M. (2016). Macrophage Metabolism Controls Tumor Blood Vessel Morphogenesis and Metastasis. Cell Metab..

[272] Colegio O.R., Chu N.-Q., Szabo A.L., Chu T., Rhebergen A.M., Jairam V., Cyrus N., Brokowski C.E., Eisenbarth S.C., Phillips G.M. (2014). Functional polarization of tumour-associated macrophages by tumour-derived lactic acid. Nature.

[273] Truong-Andrievici N., Fontaine J.-F., Kunz S., Cipriani B., Andrade-Navarro M.A., Bohn T., Bopp T. (2026). Solid tumors exploit proton-sensing GPR65 for orchestration of an immunosuppressive tumor microenvironment. Signal Transduct. Target. Ther..

[274] Feng Q., Liu Z., Yu X., Huang T., Chen J., Wang J., Wilhelm J., Li S., Song J., Li W. (2022). Lactate increases stemness of CD8+ T cells to augment anti-tumor immunity. Nat. Commun..

[275] Rofstad E.K., Mathiesen B., Kindem K., Galappathi K. (2006). Acidic extracellular pH promotes experimental metastasis of human melanoma cells in athymic nude mice. Cancer Res..

[276] Deryugina E.I., Zajac E., Juncker-Jensen A., Kupriyanova T.A., Welter L., Quigley J.P. (2014). Tissue-infiltrating neutrophils constitute the major in vivo source of angiogenesis-inducing MMP-9 in the tumor microenvironment. Neoplasia.

[277] Zhang W., Liu L., Su H., Liu Q., Shen J., Dai H., Zheng W., Lu Y., Zhang W., Bei Y. (2019). Chimeric antigen receptor macrophage therapy for breast tumours mediated by targeting the tumour extracellular matrix. Br. J. Cancer.

[278] Bergers G., Brekken R., McMahon G., Vu T.H., Itoh T., Tamaki K., Tanzawa K., Thorpe P., Itohara S., Werb Z. (2000). Matrix metalloproteinase-9 triggers the angiogenic switch during carcinogenesis. Nat. Cell Biol..

[279] Paijens S.T., Vledder A., de Bruyn M., Nijman H.W. (2021). Tumor-infiltrating lymphocytes in the immunotherapy era. Cell. Mol. Immunol..

[280] Galon J., Bruni D. (2019). Approaches to treat immune hot, altered and cold tumours with combination immunotherapies. Nat. Rev. Drug Discov..

[281] Koh J., Kim S., Woo Y.D., Song S.G., Yim J., Han B., Lim S., Ahn H.K., Mun S., Kim J.S. (2022). TCF1+PD-1+ tumour-infiltrating lymphocytes predict a favorable response and prolonged survival after immune checkpoint inhibitor therapy for non-small-cell lung cancer. Eur. J. Cancer.

[282] Im S.J., Obeng R.C., Nasti T.H., McManus D., Kamphorst A.O., Gunisetty S., Prokhnevska N., Carlisle J.W., Yu K., Sica G.L. (2023). Characteristics and anatomic location of PD-1(+)TCF1(+) stem-like CD8 T cells in chronic viral infection and cancer. Proc. Natl. Acad. Sci. USA.

[283] Simoni Y., Becht E., Fehlings M., Loh C.Y., Koo S.-L., Teng K.W.W., Yeong J.P.S., Nahar R., Zhang T., Kared H. (2018). Bystander CD8+ T cells are abundant and phenotypically distinct in human tumour infiltrates. Nature.

[284] Zeng Z., Zhang T., Zhang J., Li S., Connor S., Zhang B., Zhao Y., Wilson J., Singh D., Kulikauskas R. (2025). A minimal gene set characterizes TIL specific for diverse tumor antigens across different cancer types. Nat. Commun..

[285] Cabrita R., Lauss M., Sanna A., Donia M., Larsen M.S., Mitra S., Johansson I., Phung B., Harbst K., Vallon-Christersson J. (2020). Tertiary lymphoid structures improve immunotherapy and survival in melanoma. Nature.

[286] Parums D.V. (2024). Editorial: First Regulatory Approval for Adoptive Cell Therapy with Autologous Tumor-Infiltrating Lymphocytes (TILs)—Lifileucel (Amtagvi). Med. Sci. Monit..

[287] Medina T., Chesney J.A., Kluger H.M., Hamid O., Whitman E.D., Cusnir M., Thomas S.S., Wermke M., Domingo-Musibay E., Phan G.Q. (2025). Long-term Efficacy and Safety of Lifileucel Tumor-infiltrating Lymphocyte (TIL) Cell Therapy in Patients with Advanced Melanoma: A 5-year Analysis of the C-144-01 Study. J. Clin. Oncol..

[288] Völzke C., Ehrhardt L., Fischer L., Maul P., Wenzel C., Riabinska A., Criado-Moronati E., Dienstbier M., Hassel J., Zhang D. (2024). Clinical-scale, modular manufacturing of tumor-reactive TILs using a closed and automated culture system. Front. Immunol..

[289] Albarrán Fernández V., Martínez P.B., Granhøj J.S., Borch T.H., Donia M., Svane I.M. (2024). Biomarkers for response to TIL therapy: A comprehensive review. J. Immunother. Cancer.

[290] Sahai E., Astsaturov I., Cukierman E., DeNardo D.G., Egeblad M., Evans R.M., Fearon D., Greten F.R., Hingorani S.R., Hunter T. (2020). A framework for advancing our understanding of cancer-associated fibroblasts. Nat. Rev. Cancer.

[291] Elyada E., Bolisetty M., Laise P., Flynn W.F., Courtois E.T., Burkhart R.A., Teinor J.A., Belleau P., Biffi G., Lucito M.S. (2019). Cross-species single-cell analysis of pancreatic ductal adenocarcinoma reveals antigen-presenting cancer-associated fibroblasts. Cancer Discov..

[292] Liu Y., Sinjab A., Min J., Han G., Paradiso F., Zhang Y., Wang R., Pei G., Dai Y., Liu Y. (2025). Conserved spatial subtypes and cellular neighborhoods of cancer-associated fibroblasts revealed by single-cell spatial multi-omics. Cancer Cell.

[293] Zhang T., Ren Y., Yang P., Wang J., Zhou H. (2022). Cancer-associated fibroblasts in pancreatic ductal adenocarcinoma. Cell Death Dis..

[294] Mariathasan S. (2018). TGFβ attenuates tumour response to PD-L1 blockade by contributing to exclusion of T cells. Nature.

[295] Prenen H., Deva S., Keam B., Lindsay C.R., Lugowska I., Yang J.C., Longo F., de Miguel M., Ponz-Sarvise M., Ahn M.-J. (2024). Phase II Study to Determine the Antitumor Activity and Safety of Simlukafusp Alfa (FAP-IL2v) Combined with Atezolizumab in Esophageal Cancer. Clin. Cancer Res..

[296] Mori Y., Novruzov E., Schmitt D., Cardinale J., Watabe T., Choyke P.L., Alavi A., Haberkorn U., Giesel F.L. (2024). Clinical applications of fibroblast activation protein inhibitor positron emission tomography (FAPI-PET). npj Imaging.

[297] Mao Y., Xia Z., Xia W., Jiang P. (2024). Metabolic reprogramming, sensing, and cancer therapy. Cell Rep..

[298] Perez-Lopez R., Crispin-Ortuzar M. (2026). A multimodal murmuration for immunotherapy. Nat. Med..

[299] Xu Y., Wang Y., Fu Q., Yao Y., Zhou Y. (2026). The controversial role of metabolic reprogramming in anti-tumor therapy resistance. Discov. Oncol..

[300] Bosca L., González-Ramos S., Prieto P., Fernández-Velasco M., Mojena M., Martín-Sanz P., Alemany S. (2015). Metabolic signatures linked to macrophage polarization: From glucose metabolism to oxidative phosphorylation. Biochem. Soc. Trans..

[301] Wang X., Zhang S., Xue D., Neculai D., Zhang J. (2025). Metabolic reprogramming of macrophages in cancer therapy. Trends Endocrinol. Metab..

[302] Blagih J., Jones R.G. (2012). Polarizing macrophages through reprogramming of glucose metabolism. Cell Metab..

[303] Certo M., Niven J., Haas R., Rudzinska P., Smith J., Cucchi D., Hombrebueno J.R., Mauro C. (2024). The sedoheptulose kinase CARKL controls T-cell cytokine outputs and migration by promoting metabolic reprogramming. Discov. Immunol..

[304] Yang B., Li L., Shi D., Zhong T., Xiong H. (2025). Lactylation and antitumor immunity. Front. Immunol..

[305] Zhu Y., Ding B., Lv H., Xu H., Chen J., Xu J., Wu Q., Hao X., Hu Y., Guo J. (2026). Fasting impairs humoral immunological memory by β-hydroxybutyrate-mediated depletion of plasma cells. Immunity.

[306] Guha S., Laguette N. (2026). STING signalling as a mediator between lipid metabolism and innate immunity. Nat. Rev. Mol. Cell Biol..

[307] Lobel G.P., Jiang Y., Simon M.C. (2023). Tumor microenvironmental nutrients, cellular responses, and cancer. Cell Chem. Biol..

[308] Denk D., Karin M. (2026). Inflammation starves antitumour immunity. Nat. Cell Biol..

[309] Zhang Y.Y., Han Y., Tan Y.-T., Lu Y.-X., Ma M.-Y., Zheng Y.-Q., Chen H.-J., Fu H.-Y., Mo H.-Y., Wu Q.-N. (2026). Peritumoural adipose tissue promotes ferroptosis resistance by 3-hydroxykynurenine-mediated suppression of ferritinophagy. Nat. Cell Biol..

[310] Kang I., Theodoropoulos G., Wangpaichitr M. (2024). Targeting the kynurenine pathway: Another therapeutic opportunity in the metabolic crosstalk between cancer and immune cells. Front. Oncol..

[311] Murad J.P., Christian L., Rosa R., Ren Y., Buckley A.J., Lee E.H.J., Lopez L.S., Park A.K., Yang J., Yamaguchi Y. (2025). Solid tumour CAR-T cells engineered with fusion proteins targeting PD-L1 for localized IL-12 delivery. Nat. Biomed. Eng..

[312] Müller F., Hagen M., Wirsching A., Kharboutli S., Aigner M., Völkl S., Kretschmann S., Tascilar K., Taubmann J., Bucci L. (2026). CD19 CAR-T cells for treatment-refractory autoimmune diseases: The phase 1/2 CASTLE basket trial. Nat. Med..

[313] Ho M., Paruzzo L., Noll J.H., Stella F., Devi P., Ndeupen S., Day Y.A., Chen G.M., Cohen I.J., Ramirez-Fernandez A. (2026). CD4+ T cells mediate CAR-T cell-associated immune-related adverse events after BCMA CAR-T cell therapy. Nat. Med..

[314] Rafiq S., Brentjens R.J. (2018). Tumors evading CARs—The chase is on. Nat. Med..

[315] Chen Y., Niu S., Li Y.R., Yang L. (2025). Innovative gene engineering strategies to address tumor antigen escape in cell therapy. J. Transl. Med..

[316] Mao L., Ding L., Ding Y. (2026). Engineering the next generation of cellular therapies for solid tumors: Multi-specific armored CARs and TME reprogramming strategies. Clin. Transl. Oncol..

[317] Bhagwat A.S., Torres L., Shestova O., Shestov M., Mellors P.W., Fisher H.R., Farooki S.N., Frost B.F., Loken M.R., Gaymon A.L. (2024). Cytokine-mediated CAR T therapy resistance in AML. Nat. Med..

[318] Kumagai S., Koyama S., Itahashi K., Tanegashima T., Lin Y.-T., Togashi Y., Kamada T., Irie T., Okumura G., Kono H. (2022). Lactic acid promotes PD-1 expression in regulatory T cells in highly glycolytic tumor microenvironments. Cancer Cell.

[319] Letai A., de Thé H. (2025). Conventional chemotherapy: Millions of cures, unresolved therapeutic index. Nat. Rev. Cancer.

[320] Albelda S.M. (2024). CAR T cell therapy for patients with solid tumours: Key lessons to learn and unlearn. Nat. Rev. Clin. Oncol..

[321] Morder K.M., Nguyen M., Wilfahrt D.N., Dahmani Z.L., Burr A.B., Xie B., Morikone M., Nieves-Rosado H., Gunn W.G., Hurd D.E. (2025). Sucralose Consumption Ablates Cancer Immunotherapy Response through Microbiome Disruption. Cancer Discov..

